# Identification of Genes in *Saccharomyces cerevisiae* that Are Haploinsufficient for Overcoming Amino Acid Starvation

**DOI:** 10.1534/g3.116.037416

**Published:** 2017-02-15

**Authors:** Nancy S. Bae, Andrew P. Seberg, Leslie P. Carroll, Mark J. Swanson

**Affiliations:** * Department of Biochemistry, Arizona College of Osteopathic Medicine, Midwestern University, Glendale, Arizona 85308; † Department of Biological Science, Florida State University, Tallahassee, Florida 32306-4295; ‡ Division of Basic Medical Sciences, Mercer University School of Medicine, Macon, Georgia 31207

**Keywords:** *Saccharomyces cerevisiae*, sulfometuron methyl, amino acid starvation, general amino acid control, GCN4

## Abstract

The yeast *Saccharomyces cerevisiae* responds to amino acid deprivation by activating a pathway conserved in eukaryotes to overcome the starvation stress. We have screened the entire yeast heterozygous deletion collection to identify strains haploinsufficient for growth in the presence of sulfometuron methyl, which causes starvation for isoleucine and valine. We have discovered that cells devoid of *MET15* are sensitive to sulfometuron methyl, and loss of heterozygosity at the *MET15* locus can complicate screening the heterozygous deletion collection. We identified 138 cases of loss of heterozygosity in this screen. After eliminating the issues of the *MET15* loss of heterozygosity, strains isolated from the collection were retested on sulfometuron methyl. To determine the general effect of the mutations for a starvation response, SMM-sensitive strains were tested for the ability to grow in the presence of canavanine, which induces arginine starvation, and strains that were *MET15* were also tested for growth in the presence of ethionine, which causes methionine starvation. Many of the genes identified in our study were not previously identified as starvation-responsive genes, including a number of essential genes that are not easily screened in a systematic way. The genes identified span a broad range of biological functions, including many involved in some level of gene expression. Several unnamed proteins have also been identified, giving a clue as to possible functions of the encoded proteins.

Cells respond to changes in their external environment or alterations in internal conditions by reprogramming gene expression. In the yeast *Saccharomyces cerevisiae*, when amino acids are limiting, uncharged tRNAs accumulate, activating the Gcn2 kinase (Hinnebusch 2005). Gcn2 phosphorylates a portion of the α subunit of the general translation initiation factor, eIF-2, ultimately resulting in decreased global protein synthesis and slower translation reinitiation. While amino acids in the cell are spared by the reduction in translation, slower reinitiation favors Gcn4 translation due to several upstream open reading frames (ORFs) in its mRNA. Gcn4 is an activator of amino acid biosynthesis genes, among others (Jia *et al.* 2000; Natarajan *et al.* 2001). The increase in Gcn4 protein causes increased amino acid biosynthesis, overcoming the lack of amino acids in the cell. This response is called general amino acid control (GAAC), since its activation will lead to the increased expression of biosynthetic enzymes in the pathways of all 20 amino acids.

Genes required for GAAC have been identified using media lacking an amino acid and containing a compound that increases starvation for the omitted amino acid. Merely omitting an amino acid does not result in a robust enough response to be detected by differences in growth. Initial screens identified randomly generated mutants with reduced growth under chemically induced starvation conditions (Wolfner *et al.* 1975; Penn *et al.* 1983; Greenberg *et al.* 1986). Mutants sensitive to multiple amino acid analogs were described as having a Gcn^−^ phenotype for General Control Non-derepressible (*GCN*), in which mutant cells were unable to activate the general control pathway. More recently, genes affecting the response to amino acid starvation have been identified systematically with a collection of haploid deletion mutants. These studies used the sulfonyl urea herbicide sulfometuron methyl (SMM), which inhibits acetolactate synthase encoded by the *ILV2* gene (LaRossa and Schloss 1984; Falco and Dumas 1985), inducing starvation for isoleucine and valine. This systematic screening identified numerous coactivator complexes and subunits therein that are necessary for full activation of Gcn4 target genes (Swanson *et al.* 2003; Kim *et al.* 2005). These screens identified many of the genes involved in vesicular protein trafficking at the late endosome/multivesicular body to also be required for Gcn4 activation function (Zhang *et al.* 2008). In addition, such screening showed that the *HOM6* gene, encoding an enzyme in the pathway synthesizing homoserine, was important for normal GAAC because accumulation of the substrate for the enzyme repressed Gcn4 function (Rawal *et al.* 2014).

Based on the success of systematically screening the yeast haploid deletion mutants, and to expand upon those results, we screened the complete heterozygous deletion collection to identify genes haploinsufficient for growth under SMM-induced starvation conditions. This allows for the systematic screening of the essential genes, which has not been previously done for GAAC. We also chose to screen the heterozygous diploids of the nonessential genes. Although the haploid deletion collection has been screened to identify SMM-sensitive strains (Swanson *et al.* 2003; Kim *et al.* 2005; Zhang *et al.* 2008), some genes may not have been identified due to second-site suppressors that may arise (Huang and O’Shea 2005; Teng *et al.* 2013; Giaever and Nislow 2014). This would prevent the identification of a true positive. If the suppressor mutation is recessive, such mutations are unlikely to occur in both copies of a gene in diploids. In addition, heterozygous deletions will display little or no growth defect under normal growth conditions compared to complete deletions, so suppressor mutations should not arise and dominate a culture. SMM-sensitive strains were further characterized by testing for phenotypes under conditions of methionine and arginine starvation. These additional tests show if a gene functions in a specific amino acid pathway or if it has a broader function that affects all pathways (*e.g.*, affects GAAC).

## Materials and Methods

### Yeast strains and media

The *S. cerevisiae* heterozygous deletion collection and the BY4741 (*MAT**a**his3∆1 leu2∆0 LYS2met15∆0 ura3∆0*), BY4742 (*MATα his3∆1 leu2∆0 lys2∆0 MET15ura3∆0*) and BY4743 (*MAT**a**/MATα his3∆1/his3∆1 leu2∆0/leu2∆0 LYS2/lys2∆0 MET15/met15∆0 ura3∆0/ura3∆0*) wild-type strains were obtained from Open Biosystems (now part of GE Healthcare Dharmacon Inc.). All of the strains in the collection are of the BY4743 background, and BY4743 was used as the wild-type control for growth assays with the collection strains. We used a *GCN4/gcn4*∆ strain as our haploinsufficiency control, and this strain was generated by crossing BY4741 (*MAT**a**his3∆1 leu2∆0 LYS2met15∆0 ura3∆0*) with BY4742 *gcn4*∆ (*MATα his3∆1 leu2∆0 lys2∆0 MET15ura3∆0 gcn4∆::kanMX4*) and selecting on medium lacking methionine and lysine. A *MET15* homozygote (SY101) was generated as follows. The *GCN1/gcn1*∆ strain from the heterozygous deletion collection was sporulated and dissected (Guthrie and Fink 1991), and one of the haploid progeny SY99-4B (*MAT**a**his3∆1 leu2∆0 LYS2MET15ura3∆0*) was crossed to BY4742. Diploids were isolated by picking zygotes with a micromanipulator. They were verified as diploids by the ability to sporulate and the inability to mate.

Control strains were propagated in rich medium (YPD). Strains from the heterozygous deletion collection were grown in YPD containing 200 µg/ml G418 sulfate. Strains isolated from the heterozygous collection were subsequently grown in YPD or synthetic complete (SC) medium or SC medium lacking methionine and cysteine (SC-met-cys). All SC and SC dropout derivative media were made based on previously described methods (Adams *et al.* 1997). Per liter of medium, we used 1.7 g yeast nitrogen base without amino acids and without ammonium sulfate, 5 g ammonium sulfate, 2 g dropout mix, and 20 g glucose. Agar was added at 20 g/liter for making solid media. Our “standard” dropout mix used for the SC, synthetic complete medium lacking the amino acids isoleucine and valine (SC-ile-val) + SMM, and synthetic complete medium lacking arginine (SC-arg) + l-canavanine sulfate (CAN) media was made by mixing 2 g each of alanine, asparagine, aspartic acid, cysteine, glutamine, glutamic acid, glycine, proline, phenylalanine, tyrosine, threonine, serine, and inositol, 0.5 g adenine, and 0.2 g para-aminobenzoic acid. Media were supplemented as appropriate with the following (final concentrations listed): 0.3 mM histidine, 1 mM methionine, 1 mM lysine, 2 mM leucine, 0.5 mM isoleucine, 0.5 mM valine, 0.4 mM tryptophan, 0.2 mM uracil, and 0.5 mM arginine. For the SC-met-cys, SC-ile-val plates lacking methionine and cysteine (SC-met-cys-ile-val) + SMM, and SC-met-cys + DL-ethionine (ETH) media, the dropout mix was virtually the same except cysteine was also omitted, but arginine was included so that all of the other amino acids would be added at the same amounts as in the standard dropout mix.

Media for analyzing sensitivity to chemically induced amino acid starvation were made as follows. SMM was purchased from Chem Service, Inc. (catalog number N-13254). To make a stock solution, SMM was dissolved in DMSO at a concentration of 2 mg/ml. SMM was used in SC-ile-val plates at concentrations of 1, 2, or 3 µg/ml SMM (0.05, 0.1, and 0.15% DMSO final concentration, respectively) or in SC-met-cys-ile-val plates at concentrations of 4, 6, or 8 µg/ml SMM (0.2, 0.3, and 0.4% DMSO final concentration, respectively). ETH was purchased from Acros Organics (Thermo Fisher Scientific; catalog number AC146170100). ETH stock solutions were made at 10 mg/ml concentration in water. Plates containing ETH were made using SC-met-cys medium with ETH concentrations of 10, 15, and 20 µg/ml. CAN was purchased from Sigma-Aldrich (catalog number C9758). CAN was dissolved in water at 5 mg/ml concentration to serve as a stock solution. CAN was added to SC-arg medium at concentrations of 3 and 4 µg/ml.

### Genetic screen

The heterozygous deletion collection (∼6500 strains) was screened a single time for SMM phenotypes as follows. 96-well microtiter dishes containing the collection were thawed and duplicated using a pinning device into YPD + G418 sulfate. The strains were allowed to grow for 2 d at 30° to saturation. Wild-type (BY4743), *GCN4/gcn4*∆, and *gcn4*∆*/gcn4*∆ control strains were propagated in microtiter dishes in YPD. All strains were serially diluted manually 20-fold using 10 µl of sample diluted into 190 µl sterile water. The 20×, 400×, and 8000× dilutions were manually plated as 5-µl spots onto SC-ile-val containing 1 and 2 µg/ml SMM and SC control plates. The control strains were included on every plate. Images of the plates were taken after 3–5 d of growth at 30°. Phenotypes were scored qualitatively. All heterozygous deletion strains were compared to the wild-type strain, BY4743, and we looked for an obvious visible growth difference on the SMM media. Strains that grew to a maximum dilution where colonies were visible that were at least one dilution spot less than that of the wild-type control, taking into account any growth differences between the mutant and wild type on SC medium, were considered to be sensitive (for example, see the strains indicated in Figure 2A).

Multiple concentrations of SMM as well as phenotype observations over the course of a few days were used to ensure that phenotypes were consistent and to prevent issues of batch-to-batch media preparation. All strains displaying sensitivity to SMM compared to the wild type were isolated and seeded into new microtiter dishes for retesting. All subsequent phenotype tests for the SMM-sensitive candidates were performed and qualitatively scored at least twice.

The SMM-sensitive strains that were isolated were propagated in YPD + G418 sulfate. The SMM phenotypes of these strains were retested using 10-fold serial dilutions of samples (20 µl sample mixed into 180 µl sterile water), including control strains. Undiluted and diluted samples up to 100,000× dilution were plated as 5-µl spots on SC-ile-val medium containing 1, 2, or 3 µg/ml SMM. Images of the plates were taken after 3–5 d of growth at 30°. Strains that still displayed SMM sensitivity as described for the screen in the preceding two paragraphs (see strains in Figure 2B for examples) were isolated and grown in new 96-well microtiter dishes in YPD + G418 sulfate.

All SMM-sensitive strains were reisolated from the original collection and plated onto SC-met-cys plates. Strains that were unable to grow on SC-met-cys were considered to have lost heterozygosity at the *MET15* locus (*i.e.*, become *met15*∆*/met15*∆). These were tested for SMM sensitivity using the *met15*∆::*kanMX4/met15*∆*0* strain from the collection as a wild-type control. SMM phenotypes were assayed as in the retest described previously.

All strains identified as SMM-sensitive that were able to grow on SC-met-cys (Met^+^ phenotype) were maintained on SC-met-cys medium to prevent loss of the *MET15* allele. These strains were retested to verify their SMM sensitivity using 10-fold serial dilutions to 100,000×. The undiluted and diluted samples were plated on SC-met-cys control medium and SC-met-cys-ile-val medium containing 4, 6, or 8 µg/ml SMM. Phenotypes were assayed described previously.

After the strains that appeared SMM-sensitive due to loss of heterozygosity (LOH) were eliminated, we determined the overrepresented gene ontology (GO) categories for molecular function, biological process, and cellular component using Funspec (Robinson *et al.* 2002). A p-value cutoff of 0.001 was used.

### Subsequent screens

ETH is an analog of methionine, and as such can only be used with strains that are phenotypically Met^+^. All SMM-sensitive, Met^+^ strains maintained on SC-met-cys were tested for ETH sensitivity. Saturated cultures were serially diluted 10-fold to 100,000×. The undiluted and diluted samples were plated onto SC-met-cys control medium and SC-met-cys medium containing 10, 15, or 20 µg/ml ETH. Phenotypes on ETH were scored as described previously.

All SMM-sensitive strains, regardless of their methionine phenotype, were tested for sensitivity to CAN. Cells were propagated on YPD + G418 sulfate. Control strains were grown in YPD and 10-fold serial dilutions of saturated cultures were made to 100,000×. The diluted samples were plated onto SC control medium and SC-arg medium with 3 or 4 µg/ml CAN. Phenotypes on CAN were scored as described previously.

In order to test the effects of excess aspartate on SMM sensitivity, we added 2 g of aspartate per liter of our standard SC and SC-ile-val + 2 µg/ml SMM media, which normally contain ∼0.2 g/liter aspartate. BY4743 and *gcn4*∆*/gcn4*∆ strains were grown to saturation in YPD liquid medium. The saturated cultures were serially diluted 10-fold to 100,000×. The undiluted and diluted samples were plated onto SC, SC-ile-val + 2 µg/ml SMM, SC + 2 g/liter aspartate, and SC-ile-val + 2 µg/ml SMM + 2 g/liter aspartate plates.

### Yeast colony PCR

Prior to selection on SC-met-cys medium, all strains determined to be SMM-sensitive in the rescreen were analyzed for their *MET15* alleles using a colony PCR approach [adapted from Adams *et al.* (1997)]. Primers for PCR were as follows:

Upstream primer: 5′-GGCACGTGAAGCTGTCGATATTGG-3′; this sequence corresponds to −302 to −279, with respect to +1 of the *MET15* gene on the coding (W) strand.
ORF primer: 5′-TTCGGCAGGTTGAGAGAATTGAGG-3′; this sequence is in the ORF and corresponds to +735 to +712, with respect to +1 of the *MET15* gene on the noncoding (C) strand.
Downstream primer: 5′-AAGCCATGGGATGCTGTGTTGACC-3′; this sequence is after the stop codon and corresponds to +2775 to +2752, with respect to +1 of the *MET15* gene on the noncoding (C) strand.

Reactions included all three primers to simultaneously detect both the wild-type and *met15*∆*0* alleles. The upstream and ORF primers produce a PCR product of ∼1000 bp from the wild-type allele. The upstream and downstream primers produce a PCR product of ∼670 bp from the *met15*∆*0* allele. These two primers can also produce an ∼3000 bp fragment, but conditions of short extension time prevented significant amplification of this product.

Reaction conditions were as follows: 20 µl reaction mix (12.5 mM Tris-HCl, pH 8.5; 56 mM KCl; 0.75 mM MgCl2; 0.2 mM dNTP mix; 1 unit Taq DNA polymerase; 0.5 µM upstream primer; 0.25 µM ORF primer; and 0.25 µM downstream primer), purchased from New England Biolabs (catalog number M0267), was added to each tube. For each set of reactions, no cells were added to one tube as a negative control. To all other tubes, a small amount of cells grown on YPD were used. BY4743 cells were included in each set of reactions as a positive control for both the wild-type and *met15*∆*0* alleles. Cycling conditions were as follows: initial denaturation at 94° for 4 min; followed by 30 cycles of 94° for 1 min, 60° for 1 min, and 72° for 2 min; and a final extension at 72° for 10 min. PCR products were resolved on 1.2% agarose gels and visualized under ultraviolet light after ethidium bromide staining.

### Data availability

The authors state that all data necessary for confirming the conclusions presented in the article are represented fully within the article.

## Results and Discussion

### Screening the heterozygous deletions for growth in the presence of SMM

Early screens to identify genes involved in GAAC used randomly generated mutants that were sensitive to deprivation of each of several amino acids (Wolfner *et al.* 1975; Penn *et al.* 1983; Greenberg *et al.* 1986). More recently, screens of haploid deletion collections for sensitivity to the branched chain amino acid inhibitor SMM have been successful in identifying a plethora of genes required for the general control response (Swanson *et al.* 2003; Kim *et al.* 2005; Zhang *et al.* 2008; Rawal *et al.* 2014). To date there has been no systematic screen to identify genes required for growth during chemically induced amino acid starvation that includes all of the essential genes of yeast. One screen addressed sensitivity to SMM using heterozygous deletions including essential genes, but the screen used only 3503 strains, and the SMM containing medium also contained casamino acids (Lum *et al.* 2004), which includes isoleucine and valine. Thus, it was not a screen that would specifically identify mutants sensitive to isoleucine and valine starvation. In fact, the *GCN4/gcn4*Δ strain did not show a significant growth defect in the SMM medium with casamino acids.

**Figure 1:**
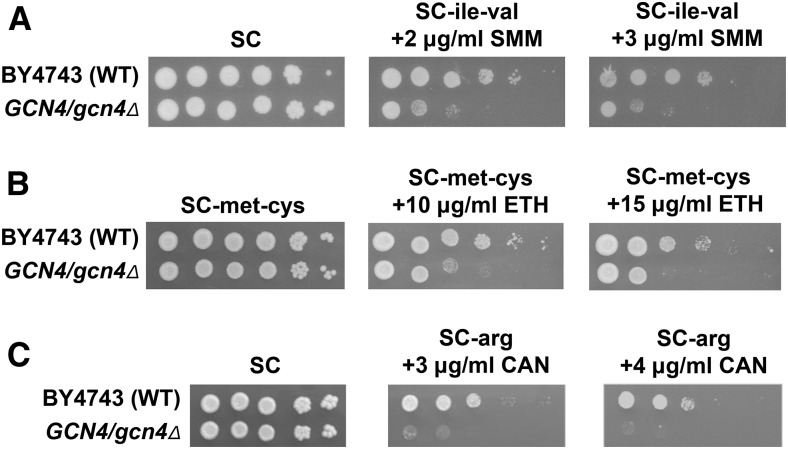
A *GCN4/gcn4*Δ strain is haploinsufficient for growth in the presence of SMM, ETH, and CAN. Ten-fold serial dilutions of wild-type BY4743 or *GCN4/gcn4*Δ cells were plated onto complete or amino acid starvation medium. (A) Cells were plated onto SC control plates and SC-ile-val plates containing SMM at the concentrations shown to induce starvation for isoleucine and valine. (B) Cells were plated onto SC-met-cys control plates (see the results section Identification of genes causing haploinsufficiency for growth on SMM for details) and SC-met-cys plates containing ETH at the concentrations shown to induce starvation for methionine. (C) Cells were plated onto SC control plates and SC-arg plates containing CAN at the concentrations shown to induce starvation for arginine.

In order to expand upon the results of the haploid deletion screens, we chose to screen the complete heterozygous deletion collection to identify genes that are haploinsufficient for growth under chemically induced amino acid starvation. In addition to being a way to systematically screen the essential genes, inclusion of the nonessential genes allows the isolation of mutants that may have been missed in haploid deletion screens due to suppressor mutations (Huang and O’Shea 2005; Teng *et al.* 2013; Giaever and Nislow 2014). We tested the *GCN4/gcn4*∆ strain for growth in the presence of amino acid analogs and biosynthetic pathway intermediates previously shown to impair the growth of yeast with mutations in genes involved in GAAC (Wolfner *et al.* 1975; Penn *et al.* 1983; Greenberg *et al.* 1986; Swanson *et al.* 2003). The *GCN4/gcn4*∆ strain was haploinsufficient for growth in the presence of SMM, the methionine analog ETH, and the arginine analog CAN (Figure 1). We were unable to utilize the histidine biosynthesis inhibitor 3-amino triazole, since the BY4743 background is *his3*Δ*/his3*Δ. The *GCN4/gcn4*Δ heterozygote did not show a haploinsufficient phenotype with either 5-methyl tryptophan (tryptophan starvation) or thialysine (lysine starvation), so they were not used (data not shown). Based on our previous success, we screened the heterozygous deletion collection with SMM. To assess the generality of each strain’s involvement in overcoming amino acid starvation, we examined additional phenotypes of the SMM-sensitive candidates using ETH and CAN.

**Figure 2:**
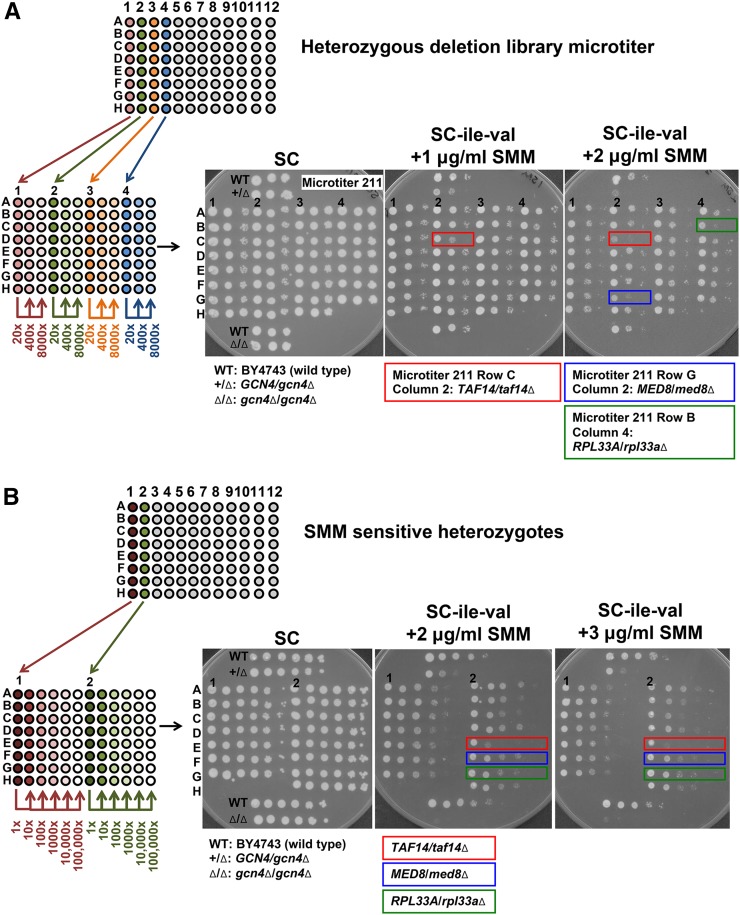
Screening for strains haploinsufficient for growth in the presence of SMM. Each panel shows a diagram of the dilution series performed as well as plates with representative data. (A) Heterozygous deletion strains from the library microtiters were transferred to microtiters with fresh YPD medium containing G418 sulfate. Every four columns of strains from each library microtiter (numbered) were transferred and diluted 20-fold per new microtiter. Two more 20-fold serial dilutions were made for each strain. For each strain, 5 µl of each dilution (20×, 400×, and 8000×) were spotted onto SC control and SC-ile-val + SMM (1 and 2 µg/ml) agar media. BY4743 (wild type), *GCN4/gcn4*∆, and *gcn4*∆*/gcn4*∆ control strains were grown in separate microtiters in YPD, and diluted samples were included on every agar plate. Plates were photographed after 3, 4, and 5 d of growth. Representative data are shown using the first four columns from microtiter #211 of the heterozygous deletion collection (the photographs show SC and SC-ile-val + 1 µg/ml SMM after 3 d of growth and the SC-ile-val + 2 µg/ml SMM after 4 d of growth). Three strains that displayed significant growth defects in the presence of SMM are indicated: *TAF14/taf14*∆ (indicated with the red boxes) on both the 1 and 2 µg/ml SMM plates, and *MED8/med8*∆ (blue box) and *RPL33A/rpl33a*∆ (green box) on the 2 µg/ml SMM plate. (B) All SMM-sensitive heterozygotes from the library were collected and organized into new microtiters. Two columns from each of the SMM-sensitive candidate microtiters (indicated by numbers 1 and 2 as an example) were used to inoculate YPD + G418 sulfate in fresh microtiters. After 2 d of growth, the strains were serially diluted 10-fold to 100,000× dilution. For each strain, 5 µl of each dilution were spotted onto SC control and SC-ile-val + SMM (1, 2, and 3 µg/ml) agar media. BY4743 (wild type), *GCN4/gcn4*∆, and *gcn4*∆*/gcn4*∆ control strains were grown in separate microtiters in YPD, and diluted samples were included on every agar plate. The photographs shown were taken after 4 d (SC and SC-ile-val + 2 µg/ml SMM) or 5 d (SC-ile-val + 3 µg/ml SMM) of growth. The three strains depicted in (A) are shown here again (the SC-ile-val + 1 µg/ml SMM plate has been omitted for clarity): *TAF14/taf14*∆ (indicated with the red boxes), *MED8/med8*∆ (blue boxes), and *RPL33A/rpl33a*∆ (green boxes).

The complete collection of yeast heterozygous deletions that includes both essential and nonessential genes was screened for strains displaying SMM sensitivity. Each strain in the collection was first grown to saturation in YPD + G418 sulfate at 30° for 2 d before being serially diluted 20-fold (up to 8000×) in sterile water in new microtiter dishes. The wild-type (BY4743), *GCN4/gcn4*∆, and *gcn4*∆*/gcn4*∆ strains were grown in YPD and served as growth controls. The dilutions for the controls and collection strains were spotted onto SC control and SC-ile-val + 1 or 2 µg/ml SMM to induce starvation. A schematic of the screening method and typical results are shown in Figure 2A. We identified 311 strains that appeared to be haploinsufficient for growth on SMM, including the *GCN4/gcn4*∆ collection strain (data not shown). These 311 SMM-sensitive strains were isolated and retested for SMM phenotypes in a new set of microtiters under the conditions described in Materials and Methods. Samples were serially diluted 10-fold and plated onto SC control and SC-ile-val + 1, 2, or 3 µg/ml SMM. A schematic of the rescreening method and typical results are shown in Figure 2B. We identified 223 mutants that reproducibly displayed sensitivity to SMM.

### SMM sensitivity of met15**∆**/met15**∆** and LOH

**Figure 3:**
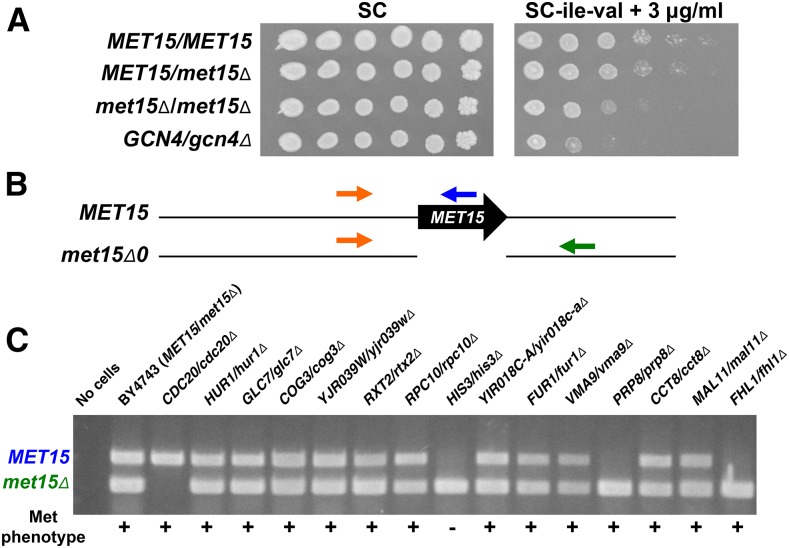
Loss of heterozygosity at the *MET15* locus and effects on SMM phenotypes. (A) Homozygous *met15*∆ strains are sensitive to SMM. The *MET15/MET15*, *MET15/met15*∆ (*met15*Δ*0*; the strain is BY4743), *met15*∆*/met15*∆ (*met15*Δ::*kanMX4/met15*Δ*0*, which is the *met15*Δ strain from the heterozygous collection), and *GCN4/gcn4*∆ strains were grown in YPD in a 96-well microtiter for 2 d at 30°. Ten-fold serial dilutions were made and 5 µl of undiluted and diluted samples were spotted onto SC control and SC-ile-val + 3 µg/ml SMM media. The plates were incubated at 30° and photographed after 3 d of growth. (B) A diagram of the *MET15* locus and oligonucleotides for yeast colony PCR are shown. The primer depicted by the orange arrow (upstream primer, see *Materials and Methods*) is upstream of the *MET15* locus and will bind to both *MET15* and *met15*∆ alleles. The primer indicated by the blue arrow (ORF primer, see *Materials and Methods*) binds to the *MET15* coding region, and it will not bind to the *met15*∆ allele. With the upstream primer, the *MET15* allele will yield a PCR product of ∼1 kbp. The primer depicted by the green arrow (downstream primer, see *Materials and Methods*) binds to a region beyond the *MET15* stop codon. This region is present in both the *MET15* and *met15*∆ alleles, but conditions for PCR were performed such that only the shorter, *met15*∆-generated PCR product was amplified. (C) A representative gel of the *MET15* locus PCR from candidate and control strains is shown. Sample names are listed above the agarose gel image. Negative (no cells) and positive (BY4743 with both the *MET15* and *met15*∆ alleles) controls were included in each gel. Methionine phenotypes are listed for each strain below the agarose gel (+, methionine prototroph; −, methionine auxotroph). Several strains show LOH at the *MET15* locus as indicated by a single band on the gel.

We next wanted to determine which of the SMM-sensitive heterozygotes were also sensitive to starvation for other amino acids to identify genes having a more general effect. We used ETH to cause a strong methionine starvation (Colombani *et al.* 1975). ETH is used with SC-met-cys medium since these two amino acids can be interconverted in yeast (Thomas and Surdin-Kerjan 1997). Among the 223 mutants that were identified as SMM-sensitive was the *met15*∆::*kanMX4/met15*∆*0* strain (Figure 3A). As expected, this strain was completely unable to grow on SC-met-cys + ETH medium (data not shown). Surprisingly, many other SMM-sensitive strains were also unable to grow on SC-met-cys + ETH medium. Subsequently, we determined that these strains were unable to grow on SC-met-cys medium without ETH, indicating that they are auxotrophic (data not shown). Previously, it was reported that the *MET15* locus exhibits an increased degree of mitotic recombination in aging yeast cells due to events occurring in the ribosomal DNA (rDNA) locus at the end of the same chromosome as *MET15* (Lindstrom *et al.* 2011). When this occurs, for a heterozygous *MET15/met15*∆ strain, the result can be LOH, causing a strain to become *MET15/MET15* or *met15*∆*/met15*∆. In research unrelated to our work, we noted that some strains from the collection, when sporulated and dissected, would result in haploid progeny that were all Met^+^ or all Met^−^ phenotypically, indicating LOH at *MET15* (data not shown). Comparing the *MET15/MET15*, *MET15/met15*∆, and *met15*∆*/met15*∆ strains showed that although the *met15*∆ homozygote is SMM-sensitive, the heterozygote and the *MET15* homozygous strain grow similarly on SMM medium (Figure 3A).

We wanted to determine whether the Met^−^ phenotypes of the SMM-sensitive mutants we identified were due to LOH or a loss of *MET15* expression by the heterozygous deletion. All 223 of the SMM-sensitive strains were reisolated from the original library microtiters and tested for growth on SC-met-cys medium. We found that 126 strains were unable to grow. Each of the 223 strains was regrown and analyzed by colony PCR using primers that simultaneously amplify the *MET15* and *met15*∆*0* alleles (Figure 3, B and C). Our results indicated that each of the 126 strains displaying the Met^−^ phenotype were homozygous for the *met15*∆*0* allele (*e.g.*, the *HIS3/his3*∆ strain in Figure 3C). Most strains that were Met^+^ were heterozygous, although three (*CDC20/cdc20*∆, *MMS4/mms4*∆, and *RRN6/rrn6*∆) were homozygous for the wild-type allele (*e.g.*, the *CDC20/cdc20*∆ strain in Figure 3C). It is important to note that LOH at the *MET15* locus is continually occurring in these strains. For example, the *FHL1/fhl1*∆ and *PRP8/prp8*∆ strains were scored as Met^+^ when the SMM-sensitive strains were grown for a phenotype test. However, when the cells were grown on a different occasion to isolate DNA for PCR, they both yielded PCR results, indicating a *met15*∆*/met15*∆ genotype (Figure 3C). These data indicate that LOH can occur during the process of a typical experiment with these strains. In all, we found that 126 strains were phenotypically Met^−^ right from the collection microtiters, which is almost 2% of the collection. Three additional strains were *MET15/MET15* by PCR. Importantly, eight of the 97 strains (∼8%) that were phenotypically Met^+^ from the collection microtiters exhibited LOH, becoming *met15*Δ*/met15*Δ by PCR during the course of their growth on YPD for the assay. Thus, we suggest that when using diploid collections, the phenotypes of *MET15/MET15*, *MET15/met15*Δ, and *met15*Δ*/met15*Δ strains should be tested under the experimental conditions to determine if there is any effect before screening the collection.

The SMM sensitivity of *met15*∆*/met15*∆ strains is most likely due to the accumulation of intermediates in the biosynthetic pathway upstream from the function of the *MET15* gene product, *O*-acetylhomoserine sulfhydrylase (Kerjan *et al.* 1986). SMM causes starvation for isoleucine and valine, leading to activation of GAAC, which induces expression of genes for amino acid biosynthesis. Homoserine is synthesized as a precursor of methionine and threonine, and threonine is converted into isoleucine. Gcn4 activates the *HOM3* and *HOM2* genes (Rawal *et al.* 2014) to generate more precursors for isoleucine synthesis, including homoserine, which is made from L-aspartate semialdehyde (ASA) by the product of the *HOM6* gene. Gcn4 also activates the *THR1* and *THR4* genes, which encode enzymes to convert homoserine into threonine. Homoserine has been shown to be toxic to yeast when it accumulates in cells such as *thr1* and *thr4* mutants (Kingsbury and McCusker 2010), and ASA accumulation dampens the GAAC response (Rawal *et al.* 2014). Homoserine is converted to methionine in several steps. The *MET2* gene encodes an enzyme that converts homoserine to *O*-acetylhomoserine. The Met15 protein is an enzyme that converts *O*-acetylhomoserine to homocysteine for further conversion to methionine. If the *MET15* gene is deleted, *O*-acetylhomoserine will build up, which hypothetically would lead to an accumulation of toxic homoserine and the GAAC inhibitor ASA. Combined, these would lead to impaired growth in the presence of SMM.

### Identification of genes causing haploinsufficiency for growth on SMM

**Figure 4:**
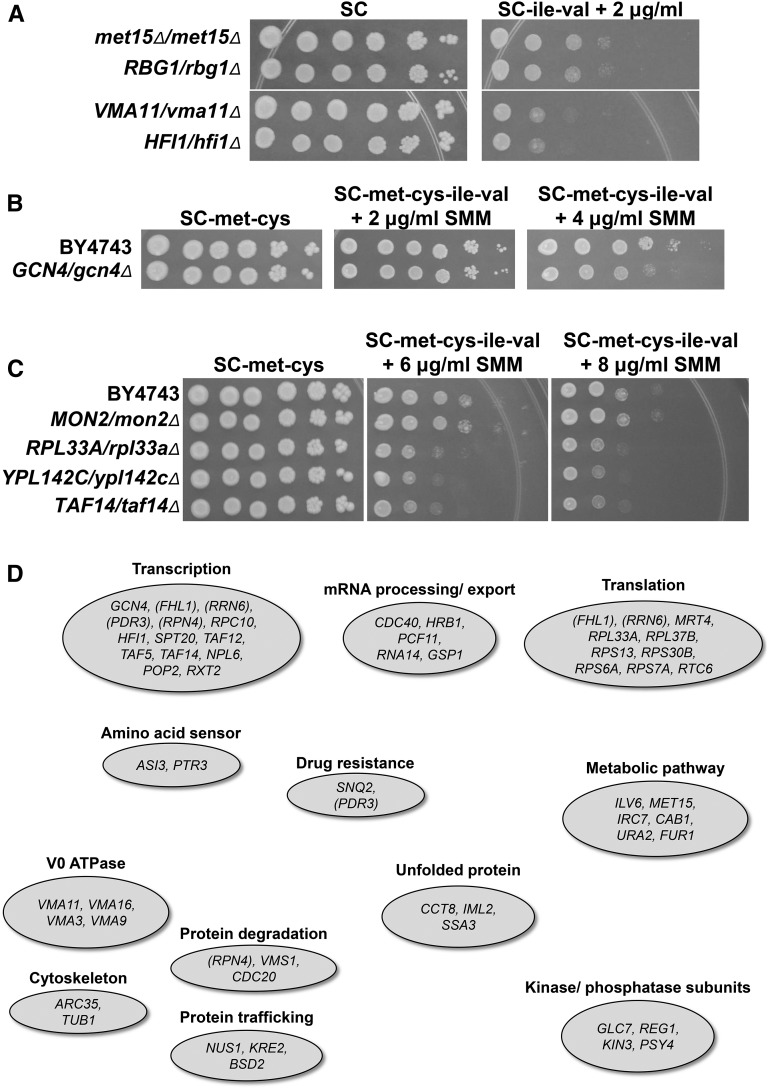
Circumventing the effects of LOH at *MET15*. (A) For all phenotypically Met^−^ deletion strains that were *met15*Δ*/met15*Δ by PCR analysis, the *met15*Δ::*kanMX4/met15*Δ*0* from the deletion strain collection was used as the wild-type control on SC-ile-val + SMM (2 µg/ml shown) containing medium. Cells were treated as in Figure 3A. As an example, the *RBG1/rbg1*Δ strain is not significantly more SMM-sensitive than the *met15*Δ::*kanMX4/met15*Δ*0* control strain. In contrast, the *VMA11/vma11*Δ and *HFI1/hfi1*Δ strains are significantly more SMM-sensitive than the control. (B) Removal of methionine and cysteine from the growth medium reduces the effectiveness of SMM. The wild-type BY4743 and the *GCN4/gcn4*Δ strains were grown and treated as in Figure 3A, except that the medium was SC-met-cys without or with 2 or 4 µg/ml SMM. In SC-met-cys, the *GCN4/gcn4*Δ strain does not display any phenotype at 2 µg/ml SMM, and a moderate phenotype at 4 µg/ml SMM (compare to Figure 1, top panels). (C) For all phenotypically Met^+^ strains, SMM sensitivity was monitored on SC-met-cys-ile-val medium without or with SMM (6 and 8 µg/ml SMM are shown). Cells were treated as in Figure 3A, except that growth media were SC-met-cys and SC-met-cys-ile-val with SMM. The *MON2/mon2*Δ strain that had originally shown an SMM-sensitive phenotype is no longer sensitive on SC-met-cys-ile-val with SMM, although the *RPL33A/rpl33a*Δ, *YPL142C/ypl142c*Δ, and *TAF14/taf14*Δ strains remain sensitive to SMM. (D) The diagram indicates the general functional categories for genes identified in the SMM-sensitivity screen as listed in Table 1. The genes from the category “Other” have been omitted.

Since the *met15*∆*0/met15*∆*0* genotype leads to SMM sensitivity, we wanted to eliminate false positives that were SMM-sensitive solely due to homozygosity for *met15*∆*0*. All strains exhibiting LOH to become *met15*∆*0/met15*∆*0* were grown on media containing SMM and compared with the *met15*∆::*kanMX4/met15*∆*0* strain, which served as the wild type (Figure 4A). A total of 98 of the 126 Met^−^ strains were observed to be no more sensitive to SMM than the *met15*∆*/met15*∆ control strain, and thus were eliminated from further study, resulting in only 28 Met^−^ strains that were SMM-sensitive due to the heterozygous deletion and not just the lack of *MET15*. These genes are listed in Table 1 and are designated as M^−^ to indicate that they are methionine auxotrophs.

**Table 1:** SMM-sensitive heterozygous deletion mutants

ORF ID	Gene	SGD Description	Phenotypes
Transcription			
YEL009C	*GCN4*	bZIP transcriptional activator of amino acid biosynthetic genes; activator responds to amino acid starvation	M^+^, E, C, v
YPR104C	*FHL1*	Regulator of ribosomal protein (RP) transcription; has forkhead associated domain that binds phosphorylated proteins; recruits coactivator Ifh1p or corepressor Crf1p to RP gene promoters	M^+^, C, i
YBL014C	*RRN6*	Component of the core factor (CF) rDNA transcription factor complex; CF is required for transcription of 35S rRNA genes by RNA polymerase I and is composed of Rrn6p, Rrn7p, and Rrn11p	M^+^, C, i
YBL005W	*PDR3*	Transcriptional activator of the pleiotropic drug resistance network; regulates expression of ATP-binding cassette (ABC) transporters	M^+^, E, C, v
YDL020C	*RPN4*	Transcription factor that stimulates expression of proteasome genes; Rpn4p levels are in turn regulated by the 26S proteasome in a negative feedback control mechanism; *RPN4* is transcriptionally regulated by various stress responses	M^+^, C, v
YHR143W-A	*RPC10*	RNA polymerase subunit ABC10-α, found in RNA pol I, II, and III	M^+^, i
YPL254W	*HFI1*	Adaptor protein required for structural integrity of the SAGA complex, a histone acetyltransferase-coactivator complex that is involved in global regulation of gene expression through acetylation and transcription functions	M^−^, C, v
YOL148C	*SPT20*	Subunit of the SAGA transcriptional regulatory complex	M^−^, C, v*^a^*
YDR145W	*TAF12*	Subunit (61/68 kD) of TFIID and SAGA complexes; involved in RNA polymerase II transcription initiation and in chromatin modification, similar to histone H2A	M^+^, E, C, i
YBR198C	*TAF5*	Subunit (90 kDa) of TFIID and SAGA complexes; involved in RNA polymerase II transcription initiation and in chromatin modification	M^+^, E, C, i
YPL129W	*TAF14*	Subunit of TFIID, TFIIF, INO80, SWI/SNF, and NuA3 complexes; involved in RNA polymerase II transcription initiation and in chromatin modification	M^+^, E, C, v
YMR091C	*NPL6*	Component of the RSC chromatin remodeling complex	M^+^, C, v
YNR052C	*POP2*	RNase of the DEDD superfamily; subunit of the Ccr4-Not complex that mediates 3′–5′ mRNA deadenylation	M^−^, C, v*^b^*
YBR095C	*RXT2*	Component of the histone deacetylase Rpd3L complex	M^+^, E, C, v
mRNA processing/export			
YDR364C	*CDC40*	Pre-mRNA splicing factor	M^+^, E, C, v*^b^*
YNL004W	*HRB1*	Poly(A^+^) RNA-binding protein; key surveillance factor for the selective export of spliced mRNAs from the nucleus to the cytoplasm	M^−^, v
YDR228C	*PCF11*	mRNA 3′ end processing factor; essential component of cleavage and polyadenylation factor IA (CF IA), involved in pre-mRNA 3′ end processing and in transcription termination	M^+^, i
YMR061W	*RNA14*	Component of the cleavage and polyadenylation factor 1 (CF1); CF1, composed of the CF1A complex (Rna14p, Rna15p, Clp1p, Pcf11p) and Hrp1, is involved in cleavage and polyadenylation of mRNA 3′ ends	M^−^, C, i
YLR293C	*GSP1*	Ran GTPase; GTP binding protein (mammalian Ranp homolog) involved in the maintenance of nuclear organization, RNA processing and transport	M^+^, C, i
Translation			
YKL009W	*MRT4*	Protein involved in mRNA turnover and ribosome assembly	M^−^, C, v
YPL143W	*RPL33A*	Ribosomal 60S subunit protein L33A; nearly identical to RPL33B	M^+^, E, C, i
YDR500C	*RPL37B*	Ribosomal 60S subunit protein L37B; required for processing of 27SB pre-rRNA and formation of stable 66S assembly intermediates; nearly identical to RPL37A	M^+^, E, C, v
YDR064W	*RPS13*	Protein component of the small (40S) ribosomal subunit	M^+^, E, i
YOR182C	*RPS30B*	Protein component of the small (40S) ribosomal subunit; identical to RPS30A	M^+^, E, C, v
YPL090C	*RPS6A*	Protein component of the small (40S) ribosomal subunit; identical to RPS6B	M^−^, v
YOR096W	*RPS7A*	Protein component of the small (40S) ribosomal subunit; nearly identical to RPS7B	M^+^, E, C, v
YPL183W-A	*RTC6*	Protein involved in translation; mutants have defects in biogenesis of nuclear ribosomes; sequence similar to prokaryotic ribosomal protein L36	M^−^, C, v
Protein kinase and phosphatase subunits			
YER133W	*GLC7*	Type 1 S/T protein phosphatase catalytic subunit; cleavage and polyadenylation factor (CPF) component	M^+^, E, C, i
YDR028C	*REG1*	Regulatory subunit of type 1 protein phosphatase Glc7p	M^+^, v
YAR018C	*KIN3*	Nonessential serine/threonine protein kinase; possible role in DNA damage response	M^−^, C, v
YBL046W	*PSY4*	Regulatory subunit of protein phosphatase PP4; presence of Psy4p in the PP4 complex is required for dephosphorylation of the histone variant H2AX during recovery from the DNA damage checkpoint	M^+^, E, C, v
Protein degradation			
YDR049W	*VMS1*	Component of a Cdc48p-complex involved in protein quality control; contributes to ER-associated degradation (ERAD) of specific substrates; forms a mitochondrially-associated complex with Cdc48p and Npl4p under oxidative stress that is required for ubiquitin-mediated mitochondria-associated protein degradation (MAD)	M^−^, C, v
YGL116W	*CDC20*	Activator of anaphase-promoting complex/cyclosome (APC/C); APC/C is required for metaphase/anaphase transition	M^+^, E, C, i
Vacuole/*V*_0_ ATPase			
YPL234C	*VMA11*	Vacuolar ATPase *V*_0_ domain subunit c′; involved in proton transport activity; N and C termini are in the vacuolar lumen	M^−^, C, v
YHR026W	*VMA16*	Subunit c′′ of the vacuolar ATPase; v-ATPase functions in acidification of the vacuole; one of three proteolipid subunits of the *V*_0_ domain	M^−^, C, v
YEL027W	*VMA3*	Proteolipid subunit c of the *V*_0_ domain of vacuolar H^+^ ATPase; required for vacuolar acidification and important for copper and iron metal ion homeostasis	M^+^, C, v
YCL005W-A	*VMA9*	Vacuolar H^+^ ATPase subunit e of the V-ATPase *V*_0_ subcomplex; essential for vacuolar acidification; involved in *V*_0_ biogenesis	M^+^, v*^a^*
Protein trafficking			
YDL193W	*NUS1*	Forms dehydrodolichyl diphosphate syntase complex with RER2 or SRT1; Nus1p may be involved in protein trafficking	M^+^, E, C, i
YDR483W	*KRE2*	α1,2-mannosyltransferase of the Golgi; involved in protein mannosylation	M^−^, C, v
YBR290W	*BSD2*	Heavy metal ion homeostasis protein; facilitates trafficking of Smf1p and Smf2p metal transporters to vacuole where they are degraded; controls metal ion transport, prevents metal hyper-accumulation, functions in copper detoxification	M^+^, C, v
Metabolic pathway			
YCL009C	*ILV6*	Regulatory subunit of acetolactate synthase; acetolactate synthase catalyzes the first step of branched-chain amino acid biosynthesis; enhances activity of the Ilv2p catalytic subunit	M^+^, v
YLR303W	*MET15*	*O*-acetyl homoserine-*O*-acetyl serine sulfhydrylase; required for Methionine and cysteine biosynthesis	M^−^, v
YFR055W	*IRC7*	β-lyase involved in the production of thiols	M^+^, v
YDR531W	*CAB1*	Pantothenate kinase, ATP:D-pantothenate 4′-phosphotransferase; catalyzes the first committed step in the universal biosynthetic pathway for synthesis of coenzyme A (CoA)	M^−^, i
YJL130C	*URA2*	Bifunctional carbamoylphosphate synthetase/aspartate transcarbamylase; catalyzes the first two enzymatic steps in the *de novo* biosynthesis of pyrimidines	M^+^, E, v
YHR128W	*FUR1*	Uracil phosphoribosyltransferase; synthesizes UMP from uracil; involved in the pyrimidine salvage pathway	M^+^, C, i
Drug resistance			
YDR011W	*SNQ2*	Plasma membrane ATP-binding cassette (ABC) transporter; multidrug transporter involved in multidrug resistance and resistance to singlet oxygen species	M^+^, v
Cytoskeleton			
YNR035C	*ARC35*	Subunit of the ARP2/3 complex; ARP2/3 is required for the motility and integrity of cortical actin patches	M^−^, C, i
YML085C	*TUB1*	α-tubulin; associates with β-tubulin (Tub2p) to form tubulin dimer, which polymerizes to form microtubules	M^+^, C, i
Amino acid sensor			
YNL008C	*ASI3*	Subunit of the nuclear inner membrane Asi ubiquitin ligase complex; acts with Asi1p and Asi2p to ensure the fidelity of SPS-sensor signaling	M^−^, C, v
YFR029W	*PTR3*	Component of the SPS plasma membrane amino acid sensor system; senses external amino acid concentration and transmits intracellular signals that result in regulation of expression of amino acid permease genes	M^−^, C, i
Unfolded protein			
YJL008C	*CCT8*	Subunit of the cytosolic chaperonin Cct ring complex; related to Tcp1p, required for the assembly of actin and tubulins *in vivo*	M^+^, C, i
YJL082W	*IML2*	Protein required for clearance of inclusion bodies; localizes to the inclusion bodies formed under protein mis-folding stress	M^+^, C, v
YBL075C	*SSA3*	ATPase involved in protein folding and the response to stress; plays a role in SRP-dependent cotranslational protein-membrane targeting and translocation	M^−^, C, v
Other			
YBR156C	*SLI15*	Subunit of the conserved chromosomal passenger complex (CPC); complex regulates kinetochore-microtubule attachments, activation of the spindle tension checkpoint, and mitotic spindle disassembly	M^+^, E, v
YNL012W	*SPO1*	Meiosis-specific prospore protein; required for meiotic spindle pole body duplication and separation	M^−^, C, v
YNL013C	*YNL013C*	Dubious open reading frame; partially overlaps the verified ORF *HEF3/YNL014W*	M^−^, C, v
YER177W	*BMH1*	14-3-3 protein, major isoform; controls proteome at posttranscriptional level, binds proteins and DNA, involved in regulation of exocytosis, vesicle transport, Ras/MAPK and rapamycin-sensitive signaling, aggresome formation, spindle position checkpoint	M^−^, v
YGL110C	*CUE3*	Protein of unknown function; has a CUE domain that binds ubiquitin, which may facilitate intramolecular monoubiquitination	M^−^, C, v
YDR516C	*EMI2*	Nonessential protein of unknown function; required for transcriptional induction of the early meiotic-specific transcription factor IME1; required for sporulation	M^+^, E, v
YGL168W	*HUR1*	Protein of unknown function; reported null mutant phenotype of hydroxyurea sensitivity may be due to effects on overlapping *PMR1* gene	M^+^, E, C, v
YGR289C	*MAL11*	High-affinity maltose transporter (α-glucoside transporter); broad substrate specificity that includes maltotriose	M^+^, E, C, v*^a^*
YBR185C	*MBA1*	Membrane-associated mitochondrial ribosome receptor	M^−^, C, v
YBR100W	*MMS4*	Subunit of structure-specific Mms4p-Mus81p endonuclease; cleaves branched DNA; involved in recombination, DNA repair, and joint molecule formation/resolution during meiotic recombination	M^+^, E, C, v
YPL142C	*YPL142C*	Dubious open reading frame; completely overlaps the verified ORF *RPL33A/YPL143W*, a component of the large (60S) ribosomal subunit	M^+^, E, C, i
YNL028W	*YNL028W*	Dubious open reading frame; partly overlaps verified ORF *KTR5/YNL029C*, a putative mannosyltransferase	M^−^, C, v
YBR221W-A	*YBR221W-A*	Putative protein of unknown function; identified by expression profiling and mass spectrometry	M^−^, v
YHL015W-A	*YHL015W-A*	Putative protein of unknown function	M^−^, v
YBR196C-A	*YBR196C-A*	Putative protein of unknown function; identified by fungal homology and RT-PCR	M^−^, C, v
YCR061W	*YCR061W*	Protein of unknown function; green fluorescent protein (GFP)-fusion protein localizes to the cytoplasm in a punctate pattern	M^+^, C, v
YBL065W	*YBL065W*	Dubious open reading frame; partially overlaps verified ORF *SEF1/YBL066C*; *YBL065W* is a nonessential gene	M^+^, E, C, v
YJR039W	*YJR039W*	Putative protein of unknown function; the authentic, nontagged protein is detected in highly purified mitochondria in high-throughput studies	M^+^, E, C, v*^a^*

Genes are grouped into general functional categories. SGD descriptions are derived from the *Saccharomyces* Genome Database. Phenotypes are: M^−^, Met^−^ (methionine auxotroph); M^+^, Met^+^ (methionine prototroph); E, ethionine sensitive (only M^+^ strains can be tested for ethionine sensitivity); C, canavanine sensitive; i, reported on SGD to be an inviable null mutation in large-scale surveys; v, reported on SGD to be a viable null deletion in large-scale surveys.

a Viability not stated on SGD, but the haploid deletion and homozygous null strains exist.

b Data in large-scale surveys include both viable and inviable phenotypes. Cells with a deletion of the *CDC40* or *POP2* genes in the S288C background that BY4743 was derived are viable.

To ensure that the Met^+^ strains from the screen were SMM-sensitive due to the heterozygous deletion and not LOH to become *met15*∆*0/met15*∆*0* occurring during the screen, the 97 Met^+^ strains of the 223 total were reisolated directly from the collection microtiters and plated onto SC-met-cys plates to maintain pressure on keeping the *MET15* allele. The *MET15/MET15* and *MET15/met15*∆*0* genotypes showed no difference in SMM sensitivity (Figure 3A), so we did not need to be concerned with LOH occurring during growth on SC-met-cys. Samples for testing SMM phenotypes were grown in liquid SC-met-cys and plated onto SC-met-cys control and SC-met-cys-ile-val + SMM plates to observe phenotypes. In leaving out the methionine and cysteine from the media, we noted that increased concentrations of SMM were required to observe similar phenotypes from the control strains compared with media containing met and cys (Figure 4B). This effect is most likely due to the presence of methionine in the SC-ile-val + SMM media. The SMM will cause a starvation for isoleucine and valine, which leads to activation of the HOM pathway. The HOM pathway produces homoserine that can be used as a precursor for methionine synthesis as well as synthesis of isoleucine and valine. Methionine represses the *MET2* gene (Baroni *et al.* 1986). So, when methionine is present and Met2 activity is lowered, less ASA and homoserine will be converted to *O*-acetylhomoserine during SMM-induced starvation. This will lead to reduced growth. However, when methionine is excluded from the medium, *MET2* is expressed when GAAC is activated, decreasing the total cellular amount of ASA and homoserine, allowing increased growth on SMM.

The heterozygotes that were able to grow on SC-met-cys were tested for SMM sensitivity on SC-met-cys-ile-val medium. In addition these plates contained increased amounts of SMM (4, 6, and 8 µg/ml; Figure 4C). Of the 97 phenotypically Met^+^ strains, only 44 displayed significant SMM sensitivity under these conditions, implying that over 50% of these isolates may have been SMM-sensitive due to LOH occurring during the course of the experiments. These strains are listed in Table 1 with the designation M^+^ to indicate they are methionine prototrophs.

Table 1 lists all of the genes that conveyed haploinsufficiency on SMM after eliminating those false positives due to LOH. They are grouped in general functional categories. With the exception of the “Other” category, these are also displayed in Figure 4D. All of the genes were analyzed for GO annotation enrichment, and the results can be found in Table 2.

**Table 2:** GO annotation enrichment

Category	Ontology	GO ID	In Category from Cluster	Intersection	Category Size	p-Value
Hydrogen ion transmembrane transporter activity	Molecular function	GO:0015078	*VMA9 VMA3 VMA16 VMA11*	4	15	1.62E−05
Amino acid binding	Molecular function	GO:0016597	*ILV6 URA2*	2	4	6.94E−04
ATP hydrolysis coupled proton transport	Biological process	GO:0015991	*VMA9 VMA3 VMA16 VMA11*	4	17	2.779E−05
Histone acetylation	Biological process	GO:0016573	*TAF5 TAF12 SPT20 TAF14 HFI1*	5	42	8.327E−05
Vacuolar acidification	Biological process	GO:0007035	*VMA9 VMA3 VMA16 VMA11*	4	26	1.61E−04
Transcription, DNA-dependent	Biological process	GO:0006351	*PDR3 RRN6 RXT2 TAF5 RPN4 TAF12 PCF11 GCN4 RPC10 NPL6 POP2 SPT20 TAF14 HFI1 FHL1*	15	540	5.83E−04
Glycogen metabolic process	Biological process	GO:0005977	*REG1 GLC7 BMH1*	3	16	6.29E−04
RNA polymerase II transcriptional preinitiation complex assembly	Biological process	GO:0051123	*TAF5 TAF12 TAF14*	3	16	6.29E−04
Regulation of carbohydrate metabolic process	Biological process	GO:0006109	*REG1 GLC7*	2	4	6.94E−04
Regulation of transcription, DNA-dependent	Biological process	GO:0006355	*PDR3 RRN6 RXT2 TAF5 RPN4 TAF12 PCF11 GCN4 NPL6 POP2 SPT20 TAF14 HFI1 FHL1*	14	507	9.70E−04
Proton transport	Biological process	GO:0015992	*VMA9 VMA3 VMA16 VMA11*	4	41	9.71E−04
Proton-transporting V-type ATPase, *V*_0_ domain	Cellular component	GO:0033179	*VMA9 VMA3 VMA16 VMA11*	4	5	6.45E−08
Vacuolar proton-transporting V-type ATPase, *V*_0_ domain	Cellular component	GO:0000220	*VMA9 VMA3 VMA16 VMA11*	4	7	4.44E−07
SLIK (SAGA-like) complex	Cellular component	GO:0046695	*TAF5 TAF12 SPT20 HFI1*	4	17	2.78E−05
Proton-transporting two-sector ATPase complex, proton-transporting domain	Cellular component	GO:0033177	*VMA3 VMA16 VMA11*	3	7	4.22E−05
SAGA complex	Cellular component	GO:0000124	*TAF5 TAF12 SPT20 HFI1*	4	20	5.52E−05
Transcription factor TFIID complex	Cellular component	GO:0005669	*TAF5 TAF12 TAF14*	3	15	5.15E−04
Intracellular	Cellular component	GO:0005622	*RPN4 VMS1 RPS13 RPL37B FUR1 MRT4 RNA14 RPS7A RPS30B RPS6A RPL33A RTC6*	12	381	7.53E−04

Overrepresented biological processes for all genes resulting in haploinsufficiency on SMM media according to the FunSpec program, using a p-value cutoff of 0.001 (Robinson *et al.* 2002). The genes identified in this screen are listed (In Category from Cluster).

It is important to note that although we used DMSO in the preparation of SMM, the maximum final concentration used in the screen of the collection was 0.1%, and the maximum final concentration for our retests was 0.4%. These amounts are less than those used in screening the homozygous deletions strains for DMSO sensitivity (1%, Gaytán *et al.* 2013; 4 and 8%, Zhang *et al.* 2013), and we did not identify any of the DMSO-sensitive mutants that were identified in those screens.

### Additional amino acid starvation assays

**Figure 5:**
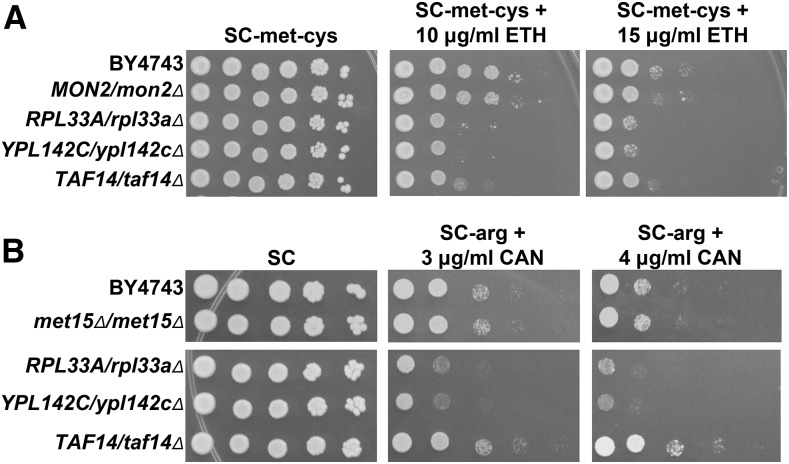
Examples of ETH and CAN haploinsufficient phenotypes. (A) ETH phenotypes of all phenotypically Met^+^ strains were tested. The cells were treated as in Figure 4B, except that they were plated onto SC-met-cys + ETH (10 and 15 µg/ml are shown). The *MON2/mon2*Δ strain grows similarly to the BY4743 wild type. The *RPL33A/rpl33a*Δ, *YPL142C/ypl142c*Δ, and *TAF14/taf14*Δ strains are sensitive to ETH. (B) All SMM-sensitive strains were tested for growth in the presence of CAN. Cells were treated as in Figure 3A, except they were plated onto SC-arg + CAN (3 and 4 µg/ml are shown). The *met15*Δ::*kanMX4/met15*Δ*0* strain is no more sensitive to CAN than the BY4743 wild type (top panels). The *RPL33A/rpl33a*Δ and *YPL142C/ypl142c*Δ strains are both CAN-sensitive but the *TAF14/taf14*Δ strain is not.

In order to characterize further the requirement for the genes identified for a robust starvation response, all of the heterozygous deletion strains identified as being SMM-sensitive were tested for their sensitivities in additional amino acid starvation conditions. Met^−^ strains cannot be tested for the ability to grow in the presence of ETH, since ETH causes starvation for methionine. The Met^+^ strains were maintained on SC-met-cys, then assayed by plating them onto SC-met-cys medium as a control and SC-met-cys + ETH to induce a strong methionine starvation. A sample of the results from this assay is shown in Figure 5A, and all ETH results can be found in Table 1. It is important to note that among the three strains that became *MET15/MET15* due to LOH (the *CDC20/cdc20*∆, *MMS4/mms4*∆, and *RRN6/rrn6*∆ strains), the *RRN6/rrn6*∆ strain was not sensitive to ETH, but both the *CDC20/cdc20*∆ and *MMS4/mms4*∆ strains were ETH sensitive. These data indicate that *MET15/MET15* homozygosity does not prevent detection of ETH sensitivity (Table 1).

All of the SMM-sensitive strains were tested for arginine starvation. Since the *met15*∆::*kanMX4/met15*∆*0* strain grew similarly to BY4743 (*MET15*/*met15*Δ*0*) on SC-arg + CAN media (Figure 5B, top panels), there was no need for propagation on selective medium to maintain the *MET15* allele. All strains were propagated on YPD before spotting to SC control or SC-arg + CAN plates. An example of the results is shown in Figure 5B, and all CAN results are indicated in Table 1.

### Genes exhibiting haploinsufficiency for amino acid starvation

After screening >6200 yeast strains heterozygous for deletions in both essential and nonessential genes, we identified 72 strains that consistently displayed sensitivity to SMM. Among these were 21 genes that are essential for viability in large-scale surveys (http://www.yeastgenome.org), and these are not easily tested for phenotypes except through haploinsufficiency screening. We also identified 51 nonessential genes. Although other screens have been performed to identify deletions that are sensitive to SMM (Swanson *et al.* 2003; Lum *et al.* 2004; Parsons *et al.* 2004; Zhang *et al.* 2008), none has ever addressed haploinsufficiency for amino acid starvation in the complete set of diploids. Since we are using heterozygotes, we are testing a decrease in gene expression, not a complete elimination of it. So, we anticipate that our results will differ from those of haploid deletion screens. Parsons *et al.* (2004) screened the *MAT***a** haploid deletion collection for strains sensitive to SMM. Their screening method used SC medium containing all amino acids and pinning onto agar medium containing just one concentration of SMM. We assayed the collection directly using serially diluted samples onto several concentrations of SMM on plates that lacked isoleucine and valine, making our assay a sensitive assay for isoleucine and valine starvation. Although some genes identified in haploid screens will not be identified using heterozygous mutants, we will be able to identify some genes that haploid deletion screens may miss due to haploids gaining second-site mutations. In heterozygous diploids, the occurrence of suppressors is less likely since there is some protein activity that lessens the effect of the deleted allele. Also, recessive suppressor mutations would need to affect both alleles of a gene in diploids, which is unlikely.

Among the SMM-sensitive strains, we identified were 44 that were phenotypically Met^+^. Within this group, we were able to screen for phenotypes on both ETH and CAN. A total of 21 strains were sensitive to all three compounds, 12 were sensitive to both SMM and CAN, four were sensitive to SMM and ETH, and seven strains were sensitive to SMM only. The 28 other SMM-sensitive strains were Met^−^, and thus they could not be tested for sensitivity to ETH since it starves for methionine. In this group, there were 21 strains that were sensitive to both SMM and CAN, and seven strains that were sensitive to SMM only.

The genes identified in our screen comprise a number of functional categories, with those involved in gene expression making up the largest portion (27 genes). In the sections that follow, we discuss the genes obtained from the screen, and they are separated into functional categories based on the information in the *Saccharomyces* Genome Database (SGD, http://www.yeastgenome.org) and the literature. We have attempted to classify the genes into categories based on their most likely connections to GAAC. In some cases, a connection to GAAC is not obvious. After the name of each gene is a parenthetical expression that states the relevant phenotypes of the gene in our screen (M^+^, methionine prototroph; M^−^, methionine auxotroph; E, sensitive to ETH; C, sensitive to CAN) and a designation of whether the gene is essential or not based on information from the SGD (i, inviable deletion in large-scale surveys; v, viable deletion in large-scale surveys). In some cases, additional names of genes are included to simplify finding important information in referenced materials.

### Genes involved in transcription

This category comprises site-specific transcription factors, activators, and repressors, as well as subunits of transcription cofactor complexes. Several transcription factors will be briefly mentioned in this section for the sake of completeness for this category, but they will be treated in more detail in other sections where they have a connection to other genes in that category.

#### GCN4 (M^+^, E, C, v):

The Gcn4 protein is a transcriptional activator of amino acid biosynthetic genes (Jia *et al.* 2000; Natarajan *et al.* 2001). It is required to overcome starvation induced by amino acid analogs and pathway intermediates (Penn *et al.* 1983; Swanson *et al.* 2003). Gcn4 has been shown to recruit several transcription cofactor complexes to target genes, and various subunits of these complexes are required to overcome amino acid deprivation (Swanson *et al.* 2003). The current screen was designed using the *GCN4/gcn4*∆ strain as a control, so it was expected that it would be identified among the positives from the heterozygous deletion collection. Also as expected, the *GCN4/gcn4*∆ heterozygote from the collection was sensitive to all three compounds used.

#### FHL1 (M^+^, C, i):

The product of the *FHL1* gene is a transcriptional regulator of ribosomal protein (RP) genes (Xiao and Grove 2009). This gene will be discussed in *Genes involved in translation*.

#### RRN6 (M^+^, C, i):

The *RRN6* gene encodes a component of the core transcription factor complex required for 35S rDNA transcription (Nogi *et al.* 1991; Keys *et al.* 1994) . This gene will be discussed in *Genes involved in translation*.

#### PDR3 (M^+^, E, C, v):

The *PDR3* gene encodes a transcription activator of the pleiotropic drug resistance network (Jungwirth and Kuchler 2006). This gene will be discussed in *Genes involved in drug resistance*.

#### RPN4 (M^+^, C, v):

The product of the *RPN4* gene is a transcription factor that activates proteasome genes (Mannhaupt *et al.* 1999). This gene will be discussed in *Genes involved in protein degradation*.

#### RPC10 (M^+^, i):

The essential *RPC10* (or *RPB12*) gene encodes a protein subunit found in all three RNA polymerase complexes (Carles *et al.* 1991). Although the effects on GAAC could stem from inefficient gene expression by any of these polymerases, Lim *et al.* (2007) showed that Gcn4 could interact with Rpc10 (Rpb12) using a Split-Ubiquitin system, suggesting that Rpc10 may behave as a cofactor for Gcn4-activated transcription. The data we present here are the first to show a functional connection between Gcn4 and Rpc10 *in vivo*. The *RPC10/rpc10*Δ strain was only sensitive to SMM, and this may be due to the fact that it may not be necessary for full transcription at every Gcn4 target gene promoter, similar to many of the other coactivators required by Gcn4 (Swanson *et al.* 2003).

#### HFI1 (M^−^, C, v):

The product of the *HFI1* (or *ADA1*) gene is a subunit of the SAGA coactivator complex that is required for complex integrity (Sterner *et al.* 1999). Haploid deletions of this gene lead to SMM sensitivity and a decrease in expression from Gcn4 reporter and *bona fide* Gcn4 target genes (*ILV2* and *HIS4*; Swanson *et al.* 2003).

#### SPT20 (M^−^, C, v):

The Spt20 (or Ada5) protein is a SAGA complex subunit (Grant *et al.* 1997), and like Hfi1 (Ada1), it is required for the integrity of the SAGA complex (Sterner *et al.* 1999). Again, similarly to *HFI1* (*ADA1*), the *spt20*Δ (*ada5*Δ) haploid strain was SMM-sensitive and displayed reduced expression from Gcn4 reporter and *bona fide* Gcn4 target genes (*ILV2* and *HIS4*; Swanson *et al.* 2003).

#### TAF12 (M^+^, E, C, i):

The essential *TAF12* (or *TAF61/68*) gene encodes a protein that is a subunit of both the TFIID and SAGA complexes (Moqtaderi *et al.* 1996; Grant *et al.* 1998). Cells with a disruption of *TAF12* by the insertion of a transposon resulted in an inability to grow in the presence of the histidine starvation compound 3-aminotriazole (3-AT) and a reduction in the expression of Gcn4 target genes during amino acid starvation (Natarajan *et al.* 1998). The effect of *TAF12/taf12*Δ on GAAC appears to be related to the function of Taf12 as part of the SAGA complex, since the insertion mutation caused a reduction in the ability of Gcn4 to interact with SAGA *in vitro*, and Gcn4 did not interact with TFIID (Drysdale *et al.* 1998).

#### TAF5 (M^+^, E, C, i):

The essential *TAF5* (or *TAF90*) gene also encodes a subunit of both the TFIID and SAGA complexes (Reese *et al.* 1994; Grant *et al.* 1998). The effect of the heterozygous deletion is likely as part of the SAGA complex, as described above for *TAF12*.

#### TAF14 (M^+^, E, C, v):

The *TAF14* (or *TFG3*) gene encodes a protein that has been found to be associated with several transcription cofactor complexes. Taf14 has been shown to be part of the TFIID, TFIIF, mediator, INO80, RSC, SWI/SNF, and NuA3 complexes (Henry *et al.* 1994; Poon *et al.* 1995; Cairns *et al.* 1996a; Du *et al.* 1998; Shen *et al.* 2003a,b; Kabani *et al.* 2005). A haploid strain deleted for the *TAF14* gene was SMM-sensitive and displayed reduced expression from Gcn4 reporter genes (Swanson *et al.* 2003). The *TAF14* heterozygous deletion was sensitive to SMM, ETH, and CAN. Since Gcn4 has been shown to interact with and recruit the mediator, RSC, and SWI/SNF complexes (Swanson *et al.* 2003), it is likely that the effects of *TAF14/taf14*Δ are due to a defect in the function of one or more of these complexes. Interestingly, the *TAF14* gene contains an intron that requires Cdc40 for its proper excision (see CDC40 in Genes involved in mRNA processing and export; Dahan and Kupiec 2004).

#### NPL6 (M^+^, C, v):

The *NPL6* (or *RSC7*) gene encodes a subunit of the RSC chromatin remodeling complex (Wilson *et al.* 2006). The RSC complex has been shown to interact with Gcn4 *in vitro*, and Gcn4 was able to recruit RSC to its target gene, *ARG1* (Swanson *et al.* 2003). There are two forms of the RSC complex containing either Rsc1 or Rsc2 (Cairns *et al.* 1996b). Analysis of *rsc1*Δ and *rsc2*Δ haploid strains indicated that although only the *rsc2*Δ strain showed weak SMM sensitivity, both subunits were required for full expression for Gcn4-mediated activation of reporter genes and *in vivo* target genes (Swanson *et al.* 2003). In addition, deletion of either *RSC1* or *RSC2* results in a decrease in TBP and RNA polymerase II recruitment to several Gcn4-activated genes (Qiu *et al.* 2004). The *NPL6/npl6*Δ strain is sensitive to SMM and CAN but not sensitive to ETH. This may be due to the fact that different Gcn4 target genes require Rsc1 and Rsc2 differentially (Swanson *et al.* 2003; Qiu *et al.* 2004).

#### POP2 (M^−^, C, v):

The *POP2* (or *CAF1*) gene encodes a protein that is part of the CCR4-NOT complex (Liu *et al.* 1998). The Pop2 and Ccr4 subunits of this complex are cytoplasmic deadenylases (Tucker *et al.* 2001). However, the CCR4-NOT complex also behaves as a coactivator of Gcn4 (Swanson *et al.* 2003). It is most likely that the *POP2/pop2*Δ strain is sensitive to starvation due to a loss of some Gcn4-coactivator function based on several lines of evidence. Deletion of any one of several CCR4-NOT complex subunits in haploids resulted in SMM sensitivity (Swanson *et al.* 2003). The *pop2*Δ (*caf1*Δ) strain exhibited reduced expression from Gcn4-driven reporter genes as well as the *bona fide* Gcn4 target genes *ILV2* and *ARG1*, which is in line with the SMM and CAN sensitivities of the heterozygous deletion strain. In haploids, deletion of *POP2* (*CAF1*) resulted in a reduced ability of Gcn4 to recruit TBP and Rpb3 (an RNA polymerase II subunit; Qiu *et al.* 2004). Finally, mutations in the *CCR4* gene that abolish its deadenylase activity do not impair the ability of cells to grow in the presence of SMM, suggesting that the mRNA degrading activities play no role in GAAC activation (data not shown).

#### RXT2 (M^+^, E, C, v):

The *RXT2* gene encodes a protein that is a subunit of the Rpd3L histone deacetylase (HDAC) complex (Carrozza *et al.* 2005; Colina and Young 2005). This complex contains the catalytic subunit Rpd3 and numerous other subunits, including Dep1, Sap30, and Ume6 (Carrozza *et al.* 2005). HDACs are typically repressors of transcription, and it is possible that a negative regulator of GAAC requires the Rpd3L complex to be repressed under conditions that induce GAAC. However, there is ample evidence that Rpd3L is also involved in gene activation (De Nadal *et al.* 2004; Sertil *et al.* 2007; Sharma *et al.* 2007; Xin *et al.* 2007; Yeheskely-Hayon *et al.* 2013). It is likely that Rpd3L may be involved in the expression of Gcn4 target genes because the subunit Dep1, in addition to Rpd3, was shown to be recruited to the *ARG1* gene in a Gcn4-dependent manner (Govind *et al.* 2010). We found that the *RXT2/rxt2*Δ strain was haploinsufficient for growth on SMM, ETH, and CAN, suggesting a broad role in GAAC. Haploid deletions of the genes encoding the subunits Sap30 and Ume6 were found to be SMM-sensitive (Parsons *et al.* 2004). This further supports the idea that Rpd3L may be playing a positive regulatory role in GAAC.

### Genes involved in mRNA processing and export

#### CDC40 (M^+^, E, C, v):

This nonessential gene encodes a pre-mRNA splicing factor. The genes identified in the current SMM sensitivity screen that contain introns according to the SGD are *YPL129W* (*TAF14*), *YNL004W* (*HRB1*), *YER133W* (*GLC7*), *YPL143W* (*RPL33A*), *YDR500C* (*RPL37B*), *YDR064W* (*RPS13*), *YOR182C* (*RPS30B*), *YPL090C* (*RPS6A*), *YOR096W* (*RPS7A*), *YJL130C* (*URA2*), *YCL005W-A* (*VMA9*), *YML085C* (*TUB1*), and *YNL012W* (*SPO1*). Any or all of these may be responsible for the phenotypes displayed by the splicing mutant. While proper intron removal of the *TAF14* (*ANC1*) mRNA depends on Cdc40 (Dahan and Kupiec 2004), the introns of *RPL25* (a RP-coding gene) and several other genes were not dependent upon Cdc40. Thus, although the RPs make up the largest group of intron-containing genes, it is most likely that the reduced activity of Cdc40 in the *CDC40/cdc40*Δ strain when starved is due to lowered levels of the Taf14 protein. The *CDC40/cdc40*Δ and *TAF14/taf14*Δ strains display the same phenotypes and sensitivity to SMM, ETH, and CAN.

#### HRB1 (M^−^, v):

The *HRB1* gene encodes a poly(A)-binding protein that is recruited to actively transcribed genes (Hurt *et al.* 2004). Hrb1 is involved in mRNA quality control, preventing the export of improperly spliced messages to allow for their degradation or recruiting Mex67 to export correct mRNAs to the cytoplasm (Hackmann *et al.* 2014). It is possible that in the *HRB1/hrb1*Δ strain, one or more of the mRNAs containing introns listed under *CDC40* may escape this surveillance, allowing aberrant mRNAs into the cytoplasm and decreasing the effective protein levels.

#### PCF11 (M^+^, i):

The essential *PCF11* gene encodes a protein component of cleavage and polyadenylation factor 1 (CF1), which is involved in mRNA 3′ end processing (Gross and Moore 2001).

#### RNA14 (M^−^, C, i):

The Rna14 protein is also a subunit of CF1 (Gross and Moore 2001). CF1 is required for cleavage and polyadenylation of the 3′ ends of mRNAs. It is made up of two components. CF1A consists of Clp1, Pcf11, Rna14, and Rna15, and CF1B is the protein Hpr1 (Gross and Moore 2001). All of the genes encoding the CF1A subunits are essential for viability. In the current screen, we identified two subunits of CF1A, Pcf11 and Rna14. Previously, the *hpr1*Δ haploid was shown to be SMM-sensitive and have decreased activation of Gcn4 reporters and *bona fide* target genes (Swanson *et al.* 2003). Taken together, the data indicate that proper CF1 function is necessary for GAAC. Although this might simply be a matter of improper processing of the 3′ ends of mRNAs, it is possible that CF1 may be involved in gene looping to facilitate transcription reinitiation, as has been shown for the Gcn4 target gene, *MET16* (Medler *et al.* 2011).

#### GSP1 (M^+^, C, i):

The essential *GSP1* gene encodes a Ran GTPase (Belhumeur *et al.* 1993). Ran GTPases are involved in the nuclear-cytoplasmic transport of proteins and RNAs, and they have been implicated in a variety of cellular functions, including replication, transcription, translation, and the cell cycle (Macara 2001). The *GSP1/gsp1*Δ strain may have a defect in any one or more of several functions that would render the cells sensitive to chemically induced starvation. The most likely effect is on the nuclear localization of Gcn4, which requires Yrb1 (Pries *et al.* 2002), a Ran GTPase–binding protein involved in nuclear import and export (Schlenstedt *et al.* 1995), and the karyopherins Srp1 and Kap95 (Pries *et al.* 2004).

### Genes involved in translation

#### FHL1 (M^+^, C, i):

The product of the *FLH1* gene is a transcriptional regulator of RP genes [reviewed in Xiao and Grove (2009)]. Deletion of *FHL1* leads to extremely slow-growing cells (Hermann-Le Denmat *et al.* 1994), but the gene has been described as essential in large-scale surveys (SGD). The *fhl1*Δ strains have reduced RP gene expression and decreased ribosomes (Rudra *et al.* 2005). In addition, the total mRNA is decreased in a *FHL1* deletion strain. Transcriptome analysis has shown that RP genes are strongly repressed when Gcn4 is highly induced in cells that are starved with 3-AT (Natarajan *et al.* 2001), and SMM also represses RP gene expression (Jia *et al.* 2000). The *FHL1/fhl1*∆ cells in this screen grew normally on control medium (data not shown) and showed sensitivity to SMM and CAN. The simplest explanation for the phenotypes we see is that the lowered level of Fhl1 protein in the heterozygote combined with the decrease in RP gene transcription when Gcn4 is induced by chemical starvation is leading to a decreased growth rate (*i.e*., not an amino acid deprivation sensitivity *per se*). It is not obvious why ETH does not have a similar effect as SMM and CAN.

#### RRN6 (M^+^, C, i):

The *RRN6* gene encodes a component of the core transcription factor complex, made up of Rrn6, Rrn7, and Rrn11 (Lalo *et al.* 1996). This complex is required for 35S rDNA transcription (Nogi *et al.* 1991; Keys *et al.* 1994) . Decreased 35S expression leads to a reduced number of ribosomes, which would lead to a situation much like that described for *FHL1/fhl1*Δ. *RRN6* and *FHL1* heterozygous deletion mutants also display similar phenotypes (SMM and CAN sensitivities). The *RRN6* heterozygous deletion was identified in this screen and not the *RRN7* or *RRN11* heterozygotes possibly due to Rrn6 being the least abundant of the three subunits (Ghaemmaghami *et al.* 2003).

#### MRT4 (M^−^, C, v):

Although the *MRT4* gene was originally identified as being involved in mRNA turnover (Zuk *et al.* 1999), it is likely that the inability to grow under conditions of amino acid deprivation is due to its role in ribosome biogenesis (Harnpicharnchai *et al.* 2001). Our reasoning is that several RP genes and genes that affect *RP* gene and rDNA expression are also sensitive to chemically induced amino acid starvation. Proteins that affect mRNA degradation do not appear to have defects in GAAC. As mentioned above for *POP2*, although a *ccr4*Δ strain is SMM-sensitive (Swanson *et al.* 2003), Ccr4 deadenylase mutants can overcome SMM (data not shown). This indicates that the RNA degradation function is not required for growth on SMM, and it is unlikely that the mRNA degradation activities of Mrt4 would be required to overcome amino acid starvation. A decrease in ribosome biogenesis in the *MRT4/mrt4*Δ strain would lead to a situation like that described for *FHL1/fhl1*Δ.

The genes remaining in this category encode RP subunits. For each gene, we primarily use the nomenclature of Planta and Mager (1998), which distinguishes duplicated genes with similar or identical proteins. We also indicate the newer, systematic designations for each protein from Ban *et al.* (2014).

#### RPL33A (M^+^, E, C, i):

*RPL33A* encodes the ribosomal 60S subunit protein L33A (eL33), which is nearly identical to the protein encoded by the *RPL33B* gene. A point mutation of *RPL33A* was shown to affect efficient pre-rRNA processing, causing defects in the biogenesis of both ribosomal subunits (Martin-Marcos *et al.* 2007).

#### RPL37B (M^+^, E, C, v):

*RPL37B* encodes the ribosomal 60S subunit protein L37B (eL37), which is nearly identical to the protein encoded by the *RPL37A* gene. In yeast, the L37 RP has been shown to be important for 60S subunit formation via pre-rRNA processing (Gamalinda *et al.* 2013).

#### RPS13 (M^+^, E, i):

*RPS13* encodes the ribosomal 40S subunit protein S13 (uS15). The S13 subunit has been shown to be involved in pre-18S rRNA processing (Ferreira-Cerca *et al.* 2005).

#### RPS30B (M^+^, E, C, v):

*RPS30B* encodes the ribosomal 40S subunit protein S30B (eS30), which is identical to the protein encoded by the *RPS30A* gene. Depletion of S30 in yeast did not lead to reduced 40S biogenesis, but it did lead to a decrease in the polysome-to-monosome ratio (Ferreira-Cerca *et al.* 2005).

#### RPS6A (M^−^, v):

*RPS6A* encodes the ribosomal 40S subunit protein S6A (eS6), which is identical to the protein encoded by the *RPS6B* gene. The S6 subunit has been shown to be involved in pre-18S rRNA processing (Ferreira-Cerca *et al.* 2005).

#### RPS7A (M^+^, E, C, v):

*RPS7A* encodes the ribosomal 40S subunit protein S7A (eS7), which is nearly identical to the protein encoded by the *RPS7B* gene. Depletion of S7 in yeast did not lead to reduced 40S biogenesis, but it did lead to a decrease in the polysome-to-80S ratio (Ferreira-Cerca *et al.* 2005).

#### RTC6 (M^−^, C, v):

*RTC6* was identified in a screen for deletions that suppress the temperature sensitivity of a *cdc13-1* mutant that lacks proper telomere capping at the nonpermissive temperature (Addinall *et al.* 2008). The *RTC6* gene encodes a protein with sequence similarity to the prokaryotic RP L36 (bL36), which is specific to bacteria, suggesting that this protein may be a mitochondrial RP (Andrade *et al.* 1997). However, mass spectrometry of proteins isolated as part of the mitochondrial ribosomal subunits using a tagged mitochondrial RP gene did not detect Rtc6 (Gan *et al.* 2002). The *rtc6*Δ (also *tae4*Δ) strain was identified in a screen for gene deletions that led to sensitivities on drugs inhibiting protein synthesis (Alamgir *et al.* 2010). Further investigation showed that *RTC6* genetically interacted with genes involved in translation and cytoplasmic ribosome biogenesis, and ribosome profiling showed that there was a reduction in cytoplasmic 40S subunits in the deletion strain. We believe that the cytoplasmic ribosome biogenesis function of Rtc6 is important for overcoming chemically induced starvation, as we have not isolated other mitochondrial RPs but we have identified several genes that affect cytoplasmic ribosomes. 

The heterozygous deletion for each of the RP genes may lead to reduced ribosome biogenesis, for example, L33A, L37B, S6A, and S13. However, it is possible that the RP genes we identified are particularly sensitive to dosage effects due to additional functions more directly related to GAAC. Particular RP subunits may be important for the detection of uncharged tRNAs in the A site of the ribosome and transfer of the signal to Gcn2 via Gcn1, as has been found for Rps10 (Lee *et al.* 2015). Specific RP subunits may be required for the translation of Gcn4 or of Gcn4-regulated mRNAs in a cell with decreased translation due to amino acid starvation–induced eIF-2α phosphorylation.

### Genes encoding protein kinase and protein phosphatase subunits

#### GLC7 (M^+^, E, C, i):

*GLC7* is an essential gene that encodes the catalytic subunit of the Protein Phosphatase Type 1 (PP1; Feng *et al.* 1991). Glc7 regulates numerous cell processes through its interaction with multiple regulatory subunits (Cannon 2010), including Reg1 (see *REG1* in the following paragraph). Glc7 was shown to act antagonistically with the Gcn2 kinase with regard to phosphorylating eIF-2α (Wek *et al.* 1992). Overexpression of *GLC7* reduced the amount of phosphorylated eIF-2α upon starvation with 3-AT, rendering cells sensitive to histidine starvation. A dominant negative allele of *GLC7*, on the other hand, was able to rescue the 3-AT sensitivity of a weakly functional allele of *GCN2*. These data appear to contradict our current study, in which the reduced amount of Glc7 that we expected in the *GLC7/glc7*Δ strain caused sensitivity to SMM, ETH, and CAN. We feel that this is due to the levels of starvation in each case. The previous study used only 10 mM 3-AT (Wek *et al.* 1992), whereas we used higher concentrations of chemicals to elicit phenotypes from heterozygous deletions. The decrease in dephosphorylation of eIF-2α in the *GLC7/glc7*Δ heterozygote combined with the degree by which our chemically induced starvation increased eIF-2α phosphorylation, may have resulted in hyper-phosphorylation of eIF-2α to a point that translation was too inhibited for the cells to produce proteins to overcome starvation.

#### REG1 (M^+^, v):

The Reg1 protein is a regulatory subunit of the PP1 Glc7 (Jiang *et al.* 2000), which was also isolated in the current screen (see *GLC7* in the preceding paragraph). It has been shown that the *reg1*Δ strain shows an increase in phosphorylation of eIF-2α upon histidine starvation with 3-AT (Cherkasova *et al.* 2010). Thus, Reg1 may be functioning similarly to Glc7, but the *REG1/reg1*Δ strain was only sensitive to SMM. It is possible that other regulatory subunits may function with Glc7 when cells are starved with ETH or CAN.

#### KIN3 (M^−^, C, v):

The nonessential *KIN3* gene encodes a serine/threonine protein kinase (Jones and Rosamond 1990). A *kin3*Δ strain was shown to be sensitive to a variety of DNA-damaging agents, suggesting a role for Kin3 in DNA damage repair (Moura *et al.* 2010). Studies on the *Aspergillus nidulans* ortholog of Kin3 show a functional interaction with microtubules and the Endosomal Sorting Complex Required for Transport (ESCRT) pathway (Govindaraghavan *et al.* 2014). This is a possibly important connection as we have identified the *TUB1/tub1*Δ strain in this screen, and multiple haploid deletions of ESCRT pathway subunits lead to SMM sensitivity (Zhang *et al.* 2008).

#### PSY4 (M^+^, E, C, v):

Based on sequence similarities, Psy4 was identified as the yeast ortholog of the mammalian R2 core regulatory subunit of the Ppp4c phosphatase, forming a complex with the regulatory Psy2 and the catalytic Pph3 subunits (Hastie *et al.* 2006). Although these genes have been linked to DNA damage repair (Vazquez-Martin *et al.* 2008), there is evidence that this phosphatase complex regulates the Gln3 transcriptional activator of nitrogen-responsive genes in response to nutrient signaling (Bertram *et al.* 2000). It is possible that its functions in response to nutrients go beyond Gln3.

### Genes involved in protein degradation

#### RPN4 (M^+^, C, v):

The *RPN4* gene encodes a transcription factor that activates genes encoding subunits of the proteasome (Mannhaupt *et al.* 1999), a complex that degrades polyubiquitinylated proteins (Finley *et al.* 2012). The function of the proteasome is important for the full expression of Gcn4 target genes (Lipford *et al.* 2005), which appears to be due to a disruption of Gcn4 binding to target promoters (Howard and Tansey 2016). Thus, it is likely the *RPN4/rpn4*Δ strain has a reduction in the expression of the proteasomal subunits. This will result in decreased promoter association, which then hinders Gcn4 target gene expression. It is interesting to note that the *PDR3/pdr3*Δ strain was also isolated in our screen, and it encodes a transcription factor that activates *RPN4* expression (Owsianik *et al.* 2002; Hahn *et al.* 2006; see *Genes involved in drug resistance below*).

#### VMS1 (M^−^, C, v):

The Vms1 protein was found to be associated with the ubiquitin-selective chaperone Cdc48 (Tran *et al.* 2011), which removes ubiquitylated proteins from complexes to target them for degradation (Xia *et al.* 2016). Cdc48 was also shown to strip transcription factors from promoters (Ndoja *et al.* 2014). In *vms1*Δ strains there is an increase in the accumulation of Cdc48-associated ubiquitinylated proteins, suggesting that Vms1 may be required to allow these proteins to be degraded (Tran *et al.* 2011). Mutation of *CDC48* was able to suppress the effects of proteasome inhibition on Gcn4 function, indicating that Cdc48 may remove ubiquitinylated Gcn4 from promoters but the ubiquitinylation is not inhibiting activation function (Howard and Tansey 2016). However, the accumulation of ubiquitinylated Gcn4 can hinder target gene expression (Lipford *et al.* 2005; Howard and Tansey 2016). Thus, we believe that in the *VMS1/vms1*Δ strain, Cdc48 will be able to evict ubiquitinylated Gcn4 from promoters but it will not be degraded, and the accumulation of ubiquitinylated Gcn4 will then hamper expression of Gcn4 target genes.

#### CDC20 (M^+^, E, C, i):

The essential *CDC20* gene encodes an activator of anaphase-promoting complex/cyclosome (APC/C), which is an E3 ubiquitin ligase that targets proteins for destruction (Finley *et al.* 2012). The primary function of the APC/C is in the regulation of cell-cycle events. The levels of Cdc20 are regulated such that Cdc20 is only able to activate the APC/C during mitosis, causing it to ubiquitinylate mitotic cyclins, Pds1p, and other anaphase inhibitors targeting them for destruction to allow anaphase progression. How the heterozygous deletion of *CDC20* leads to sensitivity to all three types of chemically induced starvation is not clear. It is possible that Cdc20, although very unstable during most of the cell cycle (Prinz *et al.* 1998), is still expressed and functional at these times. It may target a protein for degradation that is critical for cells to overcome starvation. The Gcn4 protein is regulated by ubiquitinylation and the function of the proteasome (see RPN4 in Genes involved in protein degradation). Interestingly, Cdh1, another activator of the APC/C (Finley *et al.* 2012), is able to interact with Pdr3, which is an unstable protein that is partly dependent on APC/C^Cdh1^ for its degradation (Ostapenko *et al.* 2012). The *PDR3/pdr3*Δ strain was isolated in the current screen, and it is also sensitive to all three chemicals (see PDR3 in Genes involved in drug resistance). The Pdr3 activator could also be regulated by ubiquitinylation and proteasome function, which could require Cdc20.

### Genes involved in vacuolar structure and function

Four genes encoding protein subunits of the *V*_0_ domain of the vacuolar proton-translocating ATPase (V-ATPase) were identified in our screen (Kane 2006):

*VMA3* (M^+^, C, v): This gene encodes the V-ATPase subunit c.
*VMA11* (M^−^, C, v): This gene encodes the V-ATPase subunit c′.
*VMA16* (M^−^, C, v): This gene encodes the V-ATPase subunit c′′.
*VMA9* (M^+^, v): This gene encodes the V-ATPase subunit e.

The V-ATPase of yeast functions in the acidification of the vacuole. It is comprised of two domains. The membrane-embedded *V*_0_ domain serves as a proton pore, and it consists of six subunits. The genes encoding four of these have been identified in the current screen. The *V*_1_ domain is an ATPase that is made up of eight subunits (A–H). These two domains function together to acidify the vacuole. Vacuolar acidification has been shown to be required for autophagy (Nakamura *et al.* 1997), and amino acid depletion triggers autophagy as a way to recover amino acids (Nakatogawa *et al.* 2009). Transcriptome analyses have shown that some genes involved in autophagy increase upon treatment with 3-AT (Natarajan *et al.* 2001) or SMM (Jia *et al.* 2000). Thus, it is likely that the VMA genes identified here are due to a reduction in amino acid starvation-induced autophagy due to a deficiency in vacuolar acidification. In this screen, we did not identify any of the *V*_1_ domain subunit–encoding genes, but *vma2*Δ and *vma5*Δ haploids were weakly SMM-sensitive (Zhang *et al.* 2008), and several haploid deletions of both *V*_0_ and *V*_1_ subunit–encoding genes were isolated as SMM-sensitive in a large-scale screen (Parsons *et al.* 2004). The reason we isolated these specific subunits as haploinsufficient may be solely due to differences in abundance, in which deletion of one of two copies reduces the number of functional V-ATPases. The only *V*_0_ subunits identified in other screens that tested for SMM sensitivity were *VMA6*, encoding the *V*_0_ d subunit, and *VMA16*, encoding the *V*_0_ c′′ subunit (Parsons *et al.* 2004). The reasons for the differences may be due to the use of haploids that may obtain suppressor mutations more easily, or the fact that Parsons *et al.* (2004) used complete medium with SMM.

### Genes involved in protein trafficking

#### NUS1 (M^+^, E, C, i):

The product of the essential *NUS1* gene can form a dehydrodolichyl diphosphate synthase complex with either Rer2 or Srt1 (Grabinska and Palamarczyk 2002), although neither of these were identified in the current screen, probably due to the fact that they can partly substitute for one another (Sato *et al.* 1999). Dolichol synthesis is important for *N*- and *O*-linked glycosylation of proteins (Grabinska and Palamarczyk 2002), and the *NUS1* gene is involved in protein trafficking (Yu *et al.* 2006). Yeast have a protein that resembles the mannose-6 phosphate receptor of mammals that is important for lysosomal targeting of hydrolytic enzymes (Whyte and Munro 2001). Thus, it is possible that the heterozygous deletion of *NUS1* causes a defect in protein localization within the cell, and this may result in vacuolar defects.

#### KRE2 (M^−^, C, v):

The *KRE2* gene encodes a Golgi α1,2-mannosyltransferase that is involved in protein mannosylation (Hausler *et al.* 1992; Hill *et al.* 1992). Kre2 is one of a family of related mannosyltransferase enzymes that is involved in *N*- and *O*-linked glycosylation of proteins for appropriate trafficking (Lussier *et al.* 1999). It is possible that Kre2 affects mannosylation of vacuolar hydrolytic enzymes (see Genes involved in vacuolar structure and function).

#### BSD2 (M^+^, C, v):

The *BSD2* gene encodes a protein involved in metal homeostasis via negative control over metal transporters (Liu *et al.* 1997), which is due to Bsd2 targeting the transporters to the vacuole (Liu and Culotta 1999). Bsd2 has been found to play a role in the sorting of proteins in the multi-vesicular body pathway (Hettema *et al.* 2004), which is also required for sending hydrolytic enzymes to the vacuole. Thus, the *BSD2* heterozygous deletion may disrupt vacuolar functions (see Genes involved in vacuolar structure and function).

### Genes in metabolic pathways

#### ILV6 (M^+^, v):

The *ILV6* gene encodes the regulatory subunit of acetolactate synthase, which catalyzes the first step in branched-chain amino acid biosynthesis (Cullin *et al.* 1996; Pang and Duggleby 1999). The Ilv6 protein enhances the activity of the catalytic subunit, encoded by the *ILV2* gene, which is the target of SMM (LaRossa and Schloss 1984; Falco and Dumas 1985). A decreased level of *ILV6* is expected to result in SMM sensitivity and, also as expected, the *ILV6/ilv6*Δ strain is not sensitive to either ETH or CAN.

#### MET15 (M^−^, v):

The *MET15* gene encodes the enzyme *O*-acetylhomoserine sulfhydrylase, which is involved in the synthesis of methionine (Kerjan *et al.* 1986). Since the strain isolated from the heterozygous deletion collection is actually *met15*Δ*/met15*Δ, it is not able to grow without methionine in the medium, and ETH cannot be tested. As expected, the strain is not sensitive to CAN. The reasons for the SMM-sensitive phenotype for this strain have been elaborated in SMM sensitivity of *met15*Δ/*met15*Δ and *LOH*.

#### IRC7 (M^+^, v):

The *IRC7* gene encodes a protein involved in sulfur metabolism. Irc7 has β-lyase activity, but the S288C strain has a deletion that eliminates this activity (Roncoroni *et al.* 2011), and the BY4743 background is S288C (Brachmann *et al.* 1998). Irc7 also has been described as a cysteine desulphydrase that allows cells to grow on cysteine as a sole nitrogen source, but again, the BY4743 background does not encode this function, although it could use cysteine as a sulfur source (Santiago and Gardner 2015). The *IRC7/irc7*Δ strain is only sensitive to SMM. One possibility is that Irc7 may be involved in the breakdown of SMM such that a reduction in the protein leads to increased sensitivity. An alternative is that Irc7 is involved in sulfur assimilation. SMM causes repression of the *SUL1* sulfate permease gene and the *MET3* and *MET14* genes encoding sulfur assimilation enzymes (Jia *et al.* 2000). This was not shown for 3-AT starvation, in which these same genes were induced (Natarajan *et al.* 2001). Thus, it is most likely that SMM reduces these genes specifically, possibly through diverting homoserine to isoleucine and valine synthesis, and the reduced expression is not a general property of amino acid starvation.

#### CAB1 (M^−^, i):

The essential *CAB1* gene encodes pantothenate kinase, an enzyme involved with the synthesis of coenzyme A (CoA; Olzhausen *et al.* 2009). CoA can be generated in *S. cerevisiae* from several precursors. One of these precursors is 2-keto-isovalerate (also known as 2-oxoisovalerate), which can be produced from valine by the Bat1 and Bat2 aminotransferases or from pyruvate through the Ilv proteins (Ilv2/6, Ilv5, and Ilv3). SMM media do not contain isoleucine or valine. Since SMM inhibits the Ilv2 protein and causes the production of isoleucine and valine to be decreased, 2-keto-isovalerate levels should be decreased in the cells as well. This, in turn, should lead to a decrease in the levels of pantothenate, the substrate for pantothenate kinase, which is the first to be committed and is the rate-limiting enzyme in CoA synthesis (Leonardi *et al.* 2005). Thus, the reduced pantothenate kinase activity due to the heterozygous deletion combined with reduced pantothenate precursor levels because of SMM inhibition may decrease CoA production more than either of these alone, leading to impaired cell growth. This strain could not be tested on ETH but, as expected, it was not sensitive to CAN.

#### URA2 (M^+^, E, v):

**Figure 6:**
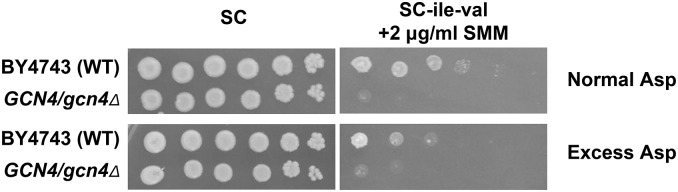
Excess aspartate exacerbates SMM-induced starvation. BY4743 wild-type cells and *gcn4*Δ*/gcn4*Δ cells treated as in Figure 3A, except that they were plated onto SC, SC-ile-val with 2 µg/ml SMM (top panels), SC with 2 g/liter aspartate added, and SC-ile-val with 2 µg/ml SMM and 2 g/liter aspartate added (bottom panels). The BY4743 cells are unaffected by addition of excess aspartate in the SC medium. When starved for isoleucine and valine, the growth of the BY4743 strain is inhibited, and the growth is made worse by the presence of the excess aspartate. The *gcn4*Δ*/gcn4*Δ strain is included as a control to show that the SMM is working.

The *URA2* gene encodes the bifunctional carbamoyl phosphate synthetase/aspartate transcarbamylase, which catalyzes the first two enzymatic steps in the *de novo* biosynthesis of pyrimidines (Denis-Duphil 1989). The *URA* genes are repressed under conditions of SMM-induced starvation (Jia *et al.* 2000) as well as 3-AT–induced starvation (Natarajan *et al.* 2001). This may be in order to spare the amino acids aspartate and glutamate, both of which are utilized by the Ura2 enzyme. With decreased Ura2 in the heterozygous deletion strain, there may be some buildup of aspartate and glutamate. Aspartate is a precursor of the HOM pathway, leading to the synthesis of methionine as well as isoleucine and valine. As mentioned earlier, an increase in homoserine and ASA, as would be expected from aspartate buildup, will cause impaired growth. The HOM pathway will be activated strongly in SMM to produce isoleucine and valine, and in ETH to produce methionine. Since arginine biosynthesis does not utilize any of those enzymes, it is understandable that the *URA2/ura2*Δ strain is not sensitive to CAN. In support of this idea, excess aspartate added to SC-ile-val + SMM medium inhibits cell growth more than SMM alone (Figure 6).

#### FUR1 (M^+^, C, i):

The *FUR1* gene encodes uracil phosphoribosyl transferase, which synthesizes uridine monophosphate (UMP) from uracil as part of the pyrimidine salvage pathway (Kern *et al.* 1990). In addition to converting uracil into UMP by Fur1, UMP can be made via *de novo* biosynthesis by the proteins encoded by the *URA* genes. The *URA3* gene encodes the enzyme for the last step in *de novo* synthesis, which converts orotidine-5′-phosphate into UMP. Deletion of *FUR1* is synthetically lethal with *ura3*∆ since cells will not be able to generate UMP (Koren *et al.* 2003). The BY4743 background is *ura3*∆*/ura3*∆, making *FUR1* critical for UMP production. Induction with SMM causes repression of *FUR1*, which may be a way for the cell to spare phosphoribosyl pyrophosphate for histidine and tryptophan synthesis when starved for amino acids (Jia *et al.* 2000). The *FUR1/fur1*∆ strain has lowered levels of Fur1 and this, together with repression due to amino acid starvation, may lead to restricted growth.

### Genes involved in drug resistance

#### SNQ2 (M^+^, v):

The *SNQ2* gene encodes an ATP-binding cassette (ABC) transporter at the plasma membrane that acts as efflux pump to eliminate toxic compounds from the cell (Jungwirth and Kuchler 2006). It is part of the pleiotropic drug resistance network, and its expression is under regulation of the Pdr1 and Pdr3 transcriptional activators. High copy *SNQ2* caused cells to become hyper-resistant to SMM, implicating Snq2 as an efflux pump for SMM (Servos *et al.* 1993). Thus, it was not surprising to find that the *SNQ2/snq2*Δ strain was SMM-sensitive. It was not sensitive to ETH or CAN.

#### PDR3 (M^+^, E, C, v):

The Pdr3 protein is one of several transcription factors that regulate genes in the pleiotropic drug resistance network (Jungwirth and Kuchler 2006). As part of this network, Pdr3 regulates the expression of ABC transporters, including *SNQ2* (DeRisi *et al.* 2000). Pdr3 is known to activate expression of *RPN4* (discussed in Genes involved in protein degradation) as part of a stress response (Owsianik *et al.* 2002; Hahn *et al.* 2006). Deletions of the *PDR3* gene or its targets result in strains that are sensitive to a variety of chemicals. Expression of *PDR3* has been observed to increase upon treatment of cells with SMM (Jia *et al.* 2000). In the current screen, the *PDR3/pdr3*Δ strain displayed sensitivity to SMM, ETH, and CAN, most likely due to a decrease in the expression of its target genes. However, a decrease in *SNQ2* and *RPN4* cannot be the reason for the sensitivities shown by the *PDR3* heterozygous deletion. The *SNQ2/snq2*Δ strain is sensitive to SMM only, and *RPN4/rpn4*Δ is sensitive to SMM and CAN, while the *PDR3/pdr3*Δ strain shows sensitivity to all three compounds. Thus, other Pdr3 targets must be involved in resistance to ETH and may partly contribute to the resistance to SMM and CAN.

### Genes involved with the cytoskeleton

#### ARC35 (M^−^, C, i):

The *ARC35* gene encodes a non-actin–related subunit of the yeast ARP2/3 complex (Winter *et al.* 1997). The ARP2/3 complex plays a role in actin polymerization, coordinating nucleation or branching of filaments (Goley and Welch 2006). Actin filaments are important for vesicular trafficking, among other things. Although it is difficult to specify a role for *ARC35* in GAAC, its effects may be connected to proper targeting of hydrolytic proteins or autophagic vesicles to the vacuole.

#### TUB1 (M^+^, C, i):

The essential *TUB1* gene encodes α-tubulin (Schatz *et al.* 1986a). Although there are two α-tubulin genes (*TUB1* and *TUB3*) encoding similar proteins that are functionally interchangeable, *TUB1* is the more highly expressed gene (Schatz *et al.* 1986b), which may be the reason it was found in the current screen and *TUB3* was not. As a component of the cytoskeleton, microtubules are important for a variety of cellular functions, including vesicular trafficking. Currently, it is difficult to pinpoint the function of *TUB1* in the response to amino acid starvation.

### Genes encoding amino acid sensor–related proteins

#### PTR3 (M^−^, C, i):

The *PTR3* gene encodes a subunit of the Ssy1-Ptr3-Ssy5 (SPS) amino acid sensor (Klasson *et al.* 1999; Forsberg and Ljungdahl 2001). The SPS sensor detects amino acids in the extracellular environment through the amino acid permease-like protein Ssy1 (Ljungdahl 2009). The cytoplasmic domain of Ssy1 interacts with Ptr3. When amino acids are sensed, Ssy1-Ptr3 signals the protease Ssy5, which also interacts with Ptr3. Ssy5 undergoes an alteration that allows it to proteolytically cleave the transcriptional activators Stp1 and Stp2, to remove a domain from each that prevents their entry into the nucleus. Once the domains are removed, the Stp proteins enter the nucleus and alter gene expression, including activating several amino acid permease genes. The Ptr3 protein appears to hold Ssy5 at the cell periphery in an inactive state, or at least to sequester it from inappropriately activating the Stp proteins. In our study, the *PTR3/ptr3*∆ strain is SMM- and CAN-sensitive, and ETH could not be tested. We believe that activation of the SPS sensor may lead to suppression of GAAC. The *PTR3* heterozygous deletion most likely allows for the cells to have some constitutive activity of the SPS sensor, since overexpression of *SSY5* has been shown to activate the sensor in the absence of amino acids in a manner that is independent of *PTR3* (Abdel-Sater *et al.* 2004). Further, it has been shown that in the presence of leucine, the *ILV* genes were repressed in a Ssy1-dependent manner (Forsberg *et al.* 2001).

#### ASI3 (M^−^, C, v):

The *ASI3* gene encodes one of three inner nuclear membrane proteins that is required to prevent the unprocessed activators Stp1 and Stp2 from entering the nucleus in the absence of external amino acids (see *PTR3* in the preceding paragraph; Zargari *et al.* 2007). The decrease in Asi3 protein in the heterozygote may be enough to allow some activation of the SPS sensor signal, by allowing the Stp proteins into the nucleus. It is unclear why *ASI3* was identified in this screen, but *ASI1* and *ASI2* were not.

### Genes that are involved in responding to unfolded proteins

#### CCT8 (M^+^, C, i):

The *CCT8* gene is essential, and it encodes one of the eight subunits of the cytosolic chaperonin Cct ring complex (Stoldt *et al.* 1996). Although numerous proteins interact with this complex for proper folding, it is required for actin and tubulin assembly *in vivo*. It is difficult to say exactly how the complex may be playing a role in GAAC. However, only the *CCT8* gene was identified in our screen, which may indicate that there is a specific substrate targeted by this complex that is important for resistance to SMM and CAN.

#### IML2 (M^+^, C, v):

Iml2 is a protein that is required for clearance of inclusion bodies, which form due to the accumulation of misfolded proteins in the cytoplasm (Moldavski *et al.* 2015). Gcn2 and Gcn4 have been shown to be required for the response to misfolded proteins accumulating in the endoplasmic reticulum (the unfolded protein response, or UPR; Patil *et al.* 2004). However, upon induction of the UPR, eIF-2α was not phosphorylated, and a *GCN4-lacZ* fusion gene and a reporter gene under Gcn4 control were both shown to be repressed (Herzog *et al.* 2013). When both GAAC and the UPR were induced simultaneously, eIF-2α was able to be phosphorylated, but there has been no report on the levels of Gcn4 or its target genes in GAAC. Under conditions where both GAAC and the UPR are simultaneously activated, the UPR may suppress GAAC to prevent the production of additional proteins that may become misfolded. It is then expected that cells would become hyper-sensitive to the amino acid starvation. It seems likely that a similar scenario would occur during cytoplasmic unfolded protein stress as well. Without clearing inclusion bodies, the response would repress GAAC, resulting in an increased sensitivity to the strong, chemically induced starvation.

#### SSA3 (M^−^, C, v):

The *SSA3* gene encodes one of a family of four heat shock chaperone (Hsp70) proteins (Werner-Washburne *et al.* 1987). Ssa3 is expressed under conditions of stress (Werner-Washburne *et al.* 1989). Hsp70 proteins are important for the efficient folding of newly made proteins, as well as refolding proteins to prevent the formation of aggregates (Hartl and Hayer-Hartl 2002). The chaperones *SSA1* and *SSA2* are expressed under normal conditions, and their levels are decreased during starvation with SMM (Jia *et al.* 2000). Thus, Ssa3 may play a more dominant role during amino acid starvation, and it may be functioning in a manner similar to Iml2 (see *IML2* in the preceding paragraph).

### Other genes

Within this group are genes with functions that we were not able to put into other categories. First, we describe those genes with known functions, followed by those that are not known but have some potentially interesting connections. Finally, we address those genes that are completely unknown.

#### SLI15 (M^+^, E, v):

The *SLI15* gene encodes a subunit of the Aurora kinase complex (or chromosomal passenger complex) that is an essential regulator of chromosome segregation, spindle checkpoint, and cytokinesis (Ruchaud *et al.* 2007). How the heterozygous deletion leads to SMM and ETH sensitivities is not immediately clear. Temperature-sensitive mutation genes involved in chromosome segregation, including *SLI15*, exhibited poly(A) mRNA accumulation in the cell nuclei and a buildup of mRNAs near transcription sites at the nonpermissive temperature (Paul and Montpetit 2016). Aneuploidy caused by loss of function in these chromosome segregation mutants could lead to the loss of genes involved in mRNA processing and export, although there has been no evidence of this, and the *SLI15/sli15*Δ strain did not exhibit LOH at *MET15* (data not shown). Thus, it is likely that the *SLI15/sli15*Δ strain is sensitive to SMM and ETH due to some issue related to export of mRNAs encoding proteins important for amino acid biosynthesis.

#### SPO1 (M^−^, C, v):

The *SPO1* gene encodes a protein that is required for meiotic spindle pole body duplication, meiotic chromosome segregation, and spore formation (Tevzadze *et al.* 2000). There is no obvious connection of this gene to amino acid starvation but it is divergently transcribed from *YNL013C* (described in the paragraph that follows), which was also identified in this screen, and both strains exhibit the same phenotypes. Since the ORFs of these two genes are separated by <200 bp, it is possible that the regulatory region of *YNL013C* overlaps with the *SPO1* ORF and is deleted in the *spo1*Δ mutation.

#### YNL013C (M^−^, C, v):

The *YNL013C* gene is listed as a dubious ORF that partly overlaps *HEF3*, which encodes a translation elongation factor (see SGD); however, we did not identify *HEF3* in our screen. There is little data to support *YNL013C* as a *bona fide* gene. However, it seems unlikely that a decrease in the Spo1 protein is causing the effects we observed. Additional work will be needed to identify which of these gene deletions results in the sensitivities we observed.

#### BMH1 (M^−^, v):

The *BMH1* and *BMH2* genes encode the yeast 14-3-3 proteins (van Heusden and Steensma 2006). Although yeast are viable when either gene is deleted, the double knockout is lethal. The 14-3-3 proteins are ubiquitous in eukaryotes, and they are involved in a variety of cell processes and in binding hundreds of other proteins. Thus, making a simple connection to GAAC without additional data seems futile.

#### CUE3 (M^−^, C, v):

The *CUE3* gene encodes a protein that contains a CUE ubiquitin-binding motif, and Cue3 was shown to bind ubiquitin (Shih *et al.* 2003). However, no function has been ascribed to this gene. It is possible that Cue3 may participate in the ubiquitinylation and/or the degradation of a factor important for GAAC, namely Gcn4 (see *RPN4* in Genes involved in protein degradation).

#### EMI2 (M^+^, E, v):

The *EMI2* gene encodes a protein of unknown function that is paralogous to Glk1, which is one of three cellular glucokinases (Walsh *et al.* 1983). A connection to GAAC is not obvious. One possibility may be that it has a role in mannose metabolism for protein glycosylation and vacuolar targeting (see Genes involved in vacuolar structure and function).

#### HUR1 (M^+^, E, C, v):

The *HUR1* gene encodes a protein of unknown function, although it was named for the fact that it is required for hydroxyurea resistance (Zewail *et al.* 2003). The *HUR1* gene overlaps with the 3′ end of the *PMR1* gene, for which deletions give similar phenotypes as *hur1*∆ (SGD) in large-scale studies, including that both have been shown to be SMM-sensitive in a large-scale screen (Parsons *et al.* 2004). We did not identify *PMR1* in the current screen. It is possible that *HUR1* encodes a protein that is important for GAAC, as the heterozygote is haploinsufficient on all three chemicals. If the results are due to *PMR1* itself, it seems likely that it is due to its function in protein trafficking as the major Golgi membrane P-type ATPase ion pump (Rudolph *et al.* 1989; see Genes involved in protein trafficking).

#### MAL11 (M^+^, E, C, v):

The *MAL11* gene encodes a high-affinity maltose transporter (Cheng and Michels 1991). However, *MAL11* is also a multi-drug resistance gene that is important for resistance to at least 56 bioactive compounds (Gossani *et al.* 2014). Thus, Mal11 is acting to eliminate drugs from the cytoplasm, and it is likely that it is in this capacity that the *MAL11* heterozygous deletion is sensitive to all three chemicals used in the current study.

#### MBA1 (M^−^, C, v):

The *MBA1* gene encodes a protein located in the mitochondrion that has been shown to be required for efficient protein insertion of both mitochondrially and nuclear-encoded proteins (Preuss *et al.* 2001). The *MBA1/mba1*Δ deletion strain is sensitive to SMM and CAN (being Met^−^, ETH could not be tested). The synthesis of isoleucine and valine occurs in the mitochondrion, as do several steps in arginine synthesis. Thus, we anticipate that decreased Mba1 may lead to a decrease in the levels of Ilv and Arg proteins in the mitochondrion, reducing levels of isoleucine, valine, and arginine in starved cells.

#### MMS4 (M^+^, E, C, v):

The protein encoded by the *MMS4* gene is a subunit of an endonuclease that cleaves recombination intermediates and is involved in recombination and repair (Bonner and Zhao 2016). There is no obvious connection of this gene to GAAC, yet the heterozygous deletion strain is sensitive to all three starvation compounds. A mutation in the *MMS4* gene was originally identified in a screen for sensitivity to the DNA-alkylating agent, methyl methanesulfonate (MMS; Xiao *et al.* 1998). Mms4 also exhibited activation function when fused to the GAL4 DNA-binding domain, although this may be fortuitous. It is interesting that MMS and 3-AT led to the induction of many of the same genes due to upregulation of Gcn4 dependent on Gcn2 (Natarajan *et al.* 2001).

#### YPL142C (M^+^, E, C, i):

*YPL142C* is a dubious ORF that almost completely overlaps the coding region of *RPL33A* (*YPL143W*). Since the heterozygous mutations of both genes cause the same set of phenotypes in this study, and both *YPL142C* and *RPL33A* are essential genes, the simplest explanation is that the *YPL142C* deletion is synonymous with a deletion of *RPL33A* (*YPL143W*). There is no compelling evidence for *YPL142C* to encode a *bona fide* protein.

#### YNL028W (M^−^, C, v):

*YNL028W* is a dubious ORF that partly overlaps the *KTR5* (*YNL029C*) gene at its 5′ end and the promoter region (SGD). KTR5 encodes a putative mannosyltransferase (Lussier *et al.* 1997). Although we did not identify the heterozygous deletion of *KTR5* in this screen, we did identify the heterozygous deletion of *KRE2*, which also encodes mannosyltransferase of the same family as Ktr5. This family of proteins is made up of nine members, although we did not identify any others in the current screen. There is no compelling data to indicate that *YNL028W* encodes a real protein, so it is possible that our data reflect a partial loss of Ktr5, which may lead to a defect in protein localization as described above for *KRE2*.

#### YBR221W-A (M−, v):

The *YBR221W-A* gene encodes a protein of unknown function.

#### YHL015W-A (M−, v):

The *YHL015W-A* gene encodes a putative protein of unknown function.

#### YBR196C-A (M−, C, v):

The *YBR196C-A* gene encodes a putative protein of unknown function.

#### YCR061W (M^+^, C, v):

The protein encoded by *YCR061W* has no known function. The protein appears to be a multi-pass membrane protein (UniProt P25639; UniProt Consortium 2015). In a large-scale survey of GFP-tagged proteins, Ycr061w showed cytoplasmic localization (Huh *et al.* 2003). The *YCR061W* gene was also found to be under control of Pdr1 (Devaux *et al.* 2001). Thus, it seems reasonable to suggest that the Ycr061w protein may be an additional drug efflux pump, similar to Snq2 (see *SNQ2* in Genes involved in drug resistance).

#### YBL065W (M^+^, E, C, v):

*YBL065W* is a dubious ORF that overlaps the promoter– and amino terminal–encoding portion of the *SEF1* (*YBL066C*) gene (SGD). The exact function of Sef1 is not known, but it encodes a protein with Zn(2)-Cys(6) binuclear cluster motif that is found in numerous transcriptional activator proteins (Groom *et al.* 1998). We did not identify *SEF1* in the current screen. Either the sensitivities to the three starvation conditions are due to a loss or partial loss of Sef1 function, or Ybl065w is a *bona fide* protein. Either way, we seem to have found a novel factor important for overcoming chemically induced amino acid starvation.

#### YJR039W (M^+^, E, C, v):

*YJR039W* encodes a protein with no known function. It is interesting that the heterozygous deletion is sensitive to all three chemicals, making it a novel gene required for GAAC.

### Conclusions

In summary, we have screened the complete heterozygous deletion collection to identify genes that are haploinsufficient for growth when starved for isoleucine and valine using SMM. Many of the heterozygotes identified also displayed sensitivity to ETH-induced methionine starvation and CAN-induced arginine starvation. Importantly, the *GCN4/gcn4*Δ strain was identified from the heterozygous deletion collection, and it was sensitive to all of the starvation conditions used, validating the screen. We also identified a number of genes that were previously identified in haploid deletion screens, such as *HFI1*, *SPT20*, and *TAF14*, although they were not necessarily expected to be haploinsufficient for amino acid starvation. The use of heterozygous deletions allowed us to identify 21 genes essential for viability that were haploinsufficient on SMM. We have identified many genes that have not been shown to have any connection to GAAC previously, and these will require further investigation to determine their molecular mechanism. We have also identified eight unnamed genes in the screen, two of which appear to be sensitive to all three chemicals, making them possible novel *GCN* genes. Before naming the unnamed genes found in this study, additional analyses to verify that they are responsible for these phenotypes seems prudent.
